# Distinct polyadenylation landscapes of diverse human tissues revealed by a modified PA-seq strategy

**DOI:** 10.1186/1471-2164-14-615

**Published:** 2013-09-11

**Authors:** Ting Ni, Yanqin Yang, Dina Hafez, Wenjing Yang, Kurtis Kiesewetter, Yoshi Wakabayashi, Uwe Ohler, Weiqun Peng, Jun Zhu

**Affiliations:** 1 National Heart Lung Blood Institute, National Institutes of Health, Genetics and Development Biology Center, 9000 Rockville Pike, Bethesda, MD, 20892, USA; 2 State Key Laboratory of Genetics Engineering & MOE Key Laboratory of Contemporary Anthropology, School of Life Sciences, Fudan University, 220 Handan Rd, Shanghai, 200433, PR China; 3 Department of Computer Science, Duke University, 101 Science Dr, Durham, NC, 27708, USA; 4 Department of Physics, The George Washington University, 21st St, NW Washington, DC, 20052, USA; 5 Institute for Genome Sciences and Policy, Duke University Medical Center, 101 Science Drive, Durham, NC, 27708, USA

## Abstract

**Background:**

Polyadenylation is a key regulatory step in eukaryotic gene expression and one of the major contributors of transcriptome diversity. Aberrant polyadenylation often associates with expression defects and leads to human diseases.

**Results:**

To better understand global polyadenylation regulation, we have developed a polyadenylation sequencing (PA-seq) approach. By profiling polyadenylation events in 13 human tissues, we found that alternative cleavage and polyadenylation (APA) is prevalent in both protein-coding and noncoding genes. In addition, APA usage, similar to gene expression profiling, exhibits tissue-specific signatures and is sufficient for determining tissue origin. A 3′ untranslated region shortening index (USI) was further developed for genes with tandem APA sites. Strikingly, the results showed that different tissues exhibit distinct patterns of shortening and/or lengthening of 3′ untranslated regions, suggesting the intimate involvement of APA in establishing tissue or cell identity.

**Conclusions:**

This study provides a comprehensive resource to uncover regulated polyadenylation events in human tissues and to characterize the underlying regulatory mechanism.

## Background

Polyadenylation is a crucial step during the maturation of pre-messenger RNAs (pre-mRNA) in eukaryotes. With a few exceptions (e.g., histone), protein coding transcripts are cleaved at their 3′ ends and subsequently polyadenylated, resulting in a characteristic poly(A) tail [1]. While 3′-end processing takes place in nucleus, it has profound effects on gene expression regulation, including transcription termination, terminal intron removal, mRNA export, translation initiation, and mRNA stability [2-5]. As one of the key regulated steps in mRNA maturation, improper cleavage and polyadenylation is often associated with expression defects and lead to human diseases [6-9].

3′-end formation of nascent transcripts is mediated by a large multi-protein complex, which in human constitutes of more than 80 proteins [9]. Several key subunits of this large complex has been characterized, including the cleavage and polyadenylation specificity factor (CPSF), cleave stimulation factor (CstF), cleavage factor I and II (CFIm, CFIIm), and poly(A) polymerase (PAP) [5]. In mammals, recruitment of the 3′-end processing complex to pre-mRNAs is mediated by a bipartite sequence element: CPSF interacts with a conserved AAUAAA or AUUAAA upstream motif, while CstF recognizes an U- or GU-rich downstream sequence element (DSE). Other factors (e.g., CFIIm and PAP) are subsequently recruited to form a functional 3′-end processing complex. Furthermore, additional sequences upstream (USEs) and/or downstream of the core elements may also play an auxiliary role in complex assembly and 3′-end formation [10]. Lastly, it has been shown that nucleosomes are preferentially depleted at the polyadenylation sites (PA sites), implying that nucleosome position might be involved in defining authentic 3′-end formation [11].

Polyadenylation is a highly regulated event and alternative polyadenylation (APA) is one of the major contributors of transcriptome diversity [12,13]. Earlier estimation suggested that approximately half of human and mouse transcripts (51.25% and 46.97%, respectively) harbor multiple poly(A) sites, leading to heterogeneous 3′ end formation [14]. A more recent survey showed that up to 80% of the human genes exhibit tissue-specific variants resulting from tandem 3′ untranslated region (3′ UTR) events [12]. Emerging evidence suggests that APA is coordinated with other regulatory events (e.g. transcriptional activity, alternative splicing and miRNA targeting) to ensure robust tissue- or cell-specific gene expression [8,12,15-18]. 3′ UTR length also plays a critical role in cell differentiation, proliferation and human diseases [8,15,16,19,20].

Global understanding of polyadenylation events in vertebrates has come primarily from cDNAs and expressed sequence tags [21]. Several genome-wide approaches have been recently developed based on tilling array [22] and sequencing based approaches [19,23-32]. Here, we present a modified polyadenylation sequencing or PA-seq strategy, allowing for precise identification of polyadenylation site at the genome scale in a cost-effective manner. By monitoring polyadenylation profiles of 13 human tissues, tissue-specific APA signatures were identified. In addition, noncoding transcripts, similar to protein-coding genes, are extensively regulated at the polyadenylation level. Lastly, we showed distinct patterns of 3′ UTR shortening/lengthening among different tissues, suggesting that APA may play a critical role in establishing tissue or cell identity. Together, the PA-seq strategy provided a comprehensive polyadenylation landscape of human transcripts across diverse tissues. It can be broadly employed to monitor polyadenylation profiles of eukaryotic transcriptomes.

## Results

### A paired-end sequencing strategy to map global polyadenylation sites

**Figure 1:**
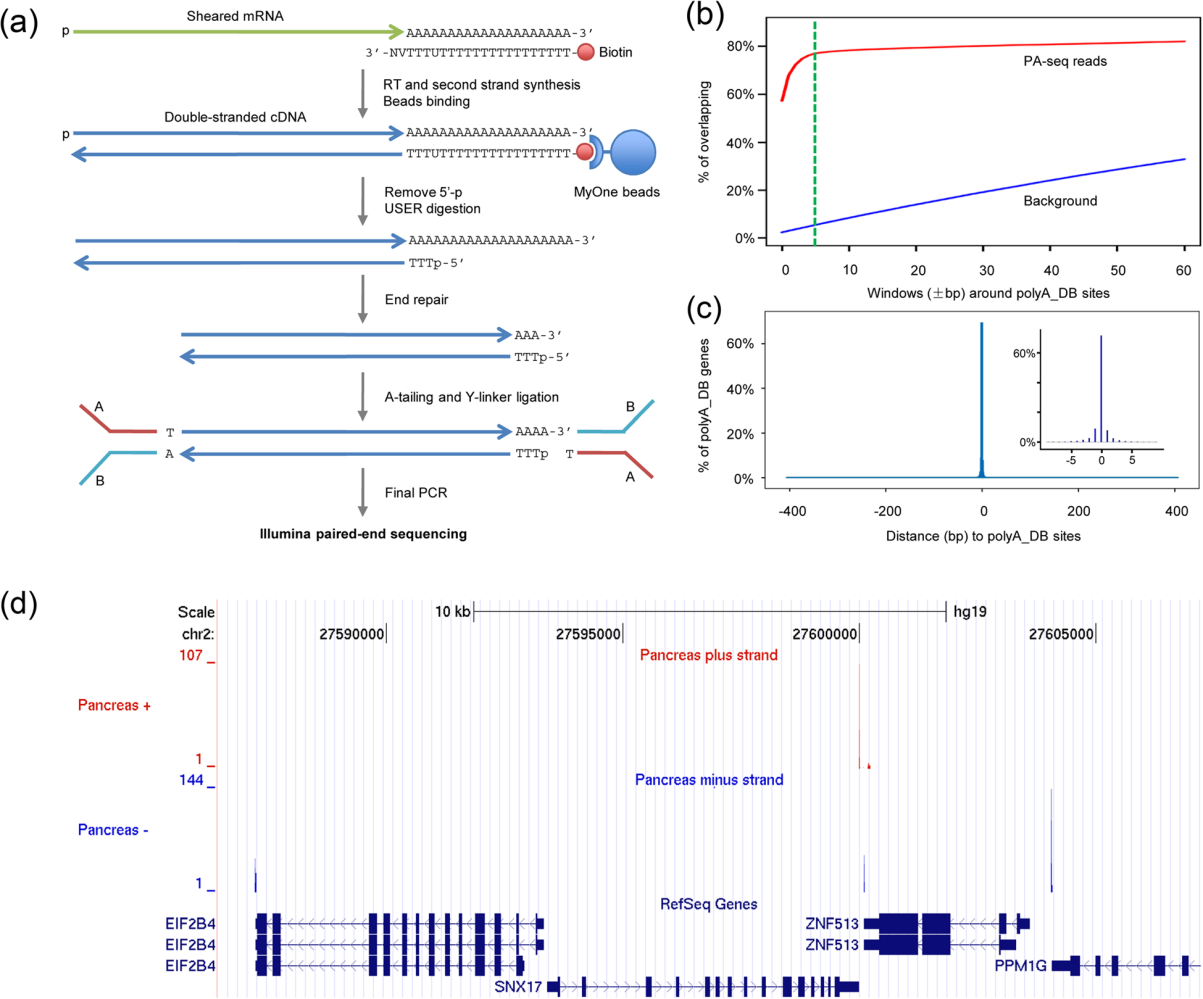
**The PA-seq strategy. (a)** Schematic diagram of the PA-seq procedure. Dual-biotin labeled DNA oligo was used for the reverse transcription. ‘U’ stands for the deoxyuracil. ‘V’ denotes any nucleotide but Thymine and ‘N’ represents any nucleotide. USER (Uracil-Specific Excision Reagent) enzyme catalyzes the excision of the uracil base in the double-stranded DNA and cleaves the abasic site to form 5′ phosphorylated (p) end. **(b)** Overlap between PA sites identified by PA-seq and those annotated in polyA_DB [14]. The background is obtained by permuting the reads within the same UTRs and then assessing the overlap. **(c)** Distance between experimentally identified PA cleavage sites and polyA_DB annotations. For genes with multiple PA sites, the shortest distance was used. **(d)** A snap view of PA cleavage sites identified by PA-seq. PA sites were separated by plus (Red) and minus (Blue) strands. Known RefSeq transcripts are shown at the bottom.

To reliably monitor genome-wide polyadenylation events, we developed a modified polyadenylation sequencing strategy or PA-seq (Figure 1a). Briefly, total RNA is fragmented and reverse transcribed with a modified oligo(dT) primer, which contains a dual biotin group at the 5′ end and a dUTP at a fixed location (the 4^th^ base from the 3′ end). After second strand synthesis, the resulting cDNA fragments are captured using streptavidin-coupled magnetic beads. Uracil-Specific Excision Reagent (USER), which recognizes and specially cleaves at the modified base (dUTP), is then used to release the cDNA fragments from beads. The resulting products are further ligated with barcoded Illumina paired-end adaptors, followed by low-cycle amplification and Illumina sequencing. Notably, the USER cleavage removes the long poly(A) stretch in cDNAs, therefore eliminating the potential complication due to these low complexity sequences. Lastly, the built-in strand specificity also allows for reliable detection of PA sites at complex genomic loci (e.g., convergent transcripts with overlapping 3′ ends).

PA-seq was employed to monitor polyadenylation profiles of 13 human tissues, including fetal and adult brains, breast, colon, heart, kidney, liver, lung, pancreas, prostate, skeletal muscle, spleen and testis. We obtained ~108 million paired 51-mer reads. Of them, 78% (~84 millions) of the read pairs can be uniquely mapped to the reference genome (Additional file 1). Another 7% of reads were mapped to multiple genomic locations, possibly due to repetitive regions in the human genome. For downstream data analyses, we focused on polyadenylation cleavage sites derived from uniquely mapped sequencing pairs.

As the initial assessment of PA-seq data, we interrogated the relative location of experimentally identified PA sites (all uniquely mapped pairs) in respect to PolyA_DB annotations [14]. In terms of coverage, ~60% of all PA sites in the PolyA_DB have concordant reads observed in our data set (Figure 1b). An additional 18% of PolyA_DB sites are within 5nt of our PA-seq data (dashed green line in Figure 1b). Conversely, more than 60% of all uniquely mapped pairs are consistent with PA sites annotated in PolyA_DB (Figure 1c). We also compared the distance between PA-seq data and RefSeq annotation, which showed similar concordant results (Additional file 2). Non-redundant read pairs were also used to avoid potential biases for highly expressed genes or due to polymerase chain reaction (PCR) artifacts. While known PA sites remained as the dominant category, distal PA sites became more prominent (Additional file 3), suggesting that novel PA sites remained to be identified. A representative genomic region with four genes showed that the majority of the reads overlapped with the annotated PA sites (Figure 1d). Together, these results strongly suggested that PA-seq is a reliable approach for direct monitoring of genome-wide polyadenylation events.

To examine whether the PA-seq results reflect relative gene expression level, the tag counts of individual genes were compared with the available microarray-based expression datasets of the same tissue origin. With minimal normalization of both the array and the sequence data, PA-seq and array expression profiles were considerably correlated (R = 0.64, Additional file 4). This result was comparable with the correlation observed between typical RNA sequencing (RNA-seq) and array-based methods [33,34]. PA-seq expression profile was also correlated with RNA-seq (R = 0.76, Additional file 5). Therefore, the read count generated with the PA-seq approach can potentially be used to reflect transcript abundance.

### Prevalent APA in protein-coding and noncoding transcripts

**Figure 2:**
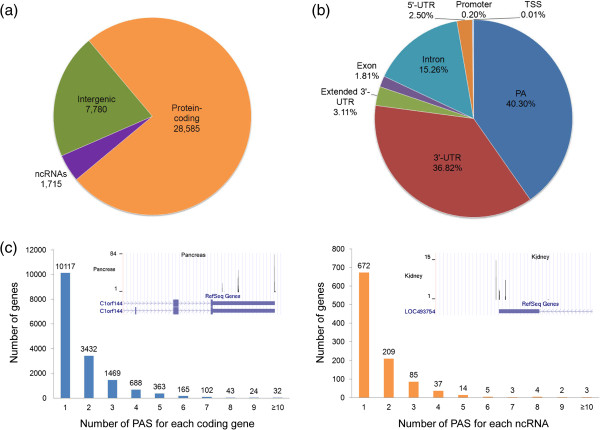
**The genomic location of PA clusters identified by PA-seq. (a)** The genomic location of all PA clusters identified. The number of PA clusters for each subcategory is shown. **(b)** The location of PA clusters in protein-coding genes based on RefSeq annotation. **(c)** The number of PA clusters detected for protein-coding genes (left panel) and noncoding RNAs (right panel), respectively. The inset in each panel shows a representative locus with known annotations as well as PA clusters identified.

Next, we applied F-seq, a feature density estimator [34,35], to compute PA cluster based on uniquely mapped read pairs. The PA-seq data from 13 tissues were combined so that a unified peak-calling scheme can be applied. To filter out any potential nonspecific priming events, we removed PA clusters with 15 or more ‘A’ in the 20 nt region downstream of peak mode (the most frequent position in the cluster). With a minimum of 50 tags, 38,080 discrete PA clusters were identified, which cover 16,435 coding genes and 1,034 non-coding RNAs (ncRNAs) based on RefSeq annotation. Overall, there are 28,585 PA clusters within the protein-coding loci, of which 38% have two or more PA clusters (Figure 2a,c). Consistent with a previous estimation [14], each coding gene has on average 1.74 PA clusters. In addition, we identified 1,715 PA clusters covering 1,034 annotated ncRNAs (or 1.66 PA cluster per ncRNA), and 35% ncRNAs have two or more PA clusters (Figure 2c, right panel). These results strongly indicated that ncRNAs, similar to the coding transcripts, are extensively regulated by alternative polyadenylation. The rest of the PA clusters (7714, ~20%) are distant (> 500 bp) from any annotated regions. Although some of them might represent distant PA sites of known genes, others are likely derived from novel transcripts in intergenic regions. The distribution of these distant PA clusters largely followed the genome-wide trend (Additional file 6). Furthermore, we found that majority (~87%) of PA clusters are employed in 5 or more tissues, including 35.53% of the clusters that can be detected in all 13 tissues (Additional file 7). The distribution of the distant PA clusters (7714) showed a similar trend: majority (~90%) of them are employed in 5 or more tissues, and 17.92% of the PA clusters can be detected in all 13 tissues (Additional file 7). These data suggested that genic and intergenic PA sites are relatively ubiquitously employed, while a considerable proportion (10.11 ~ 13.36%) may exhibit tissue-restricted usage (Additional file 7). Overall, our data serve as a unique resource to uncover novel PA sites in known genes and/or novel transcripts.

For the protein-coding PA clusters, their relative locations within respective genes were determined (Figure 2b). 40.3% of the clusters are overlapped with known polyadenylation sites, 36.82% and 3.11% clusters are located in the annotated and extended 3′ UTR regions (500 bp downstream of the most proximal 3′ PA sites), respectively. These alternative PA sites are expected to modulate the length of 3′ UTRs and may affect the stability, localization and/or translation of the corresponding transcripts [14,23]. In addition, a considerable proportion (15.26%) of the PA clusters fall into introns, suggesting the potential coupling of alternative splicing and polyadenylation [12]. A small subset of the PA clusters is mapped to exons (1.81%) as well as the upstream regions of the transcripts (5′ UTR: 2.5%; transcription start sites: 0.01%; promoter: 0.2%; Figure 2b). These 5′ proximal PA clusters are interesting and may have resulted from transcriptional read-through [36], enhancer RNAs [37,38] or other novel mechanisms [39]. Together, our data support the notion that 3′-end formation in the human transcriptomes is much more complex than previously appreciated [23,30,36].

### Validation of novel PA clusters

**Figure 3:**
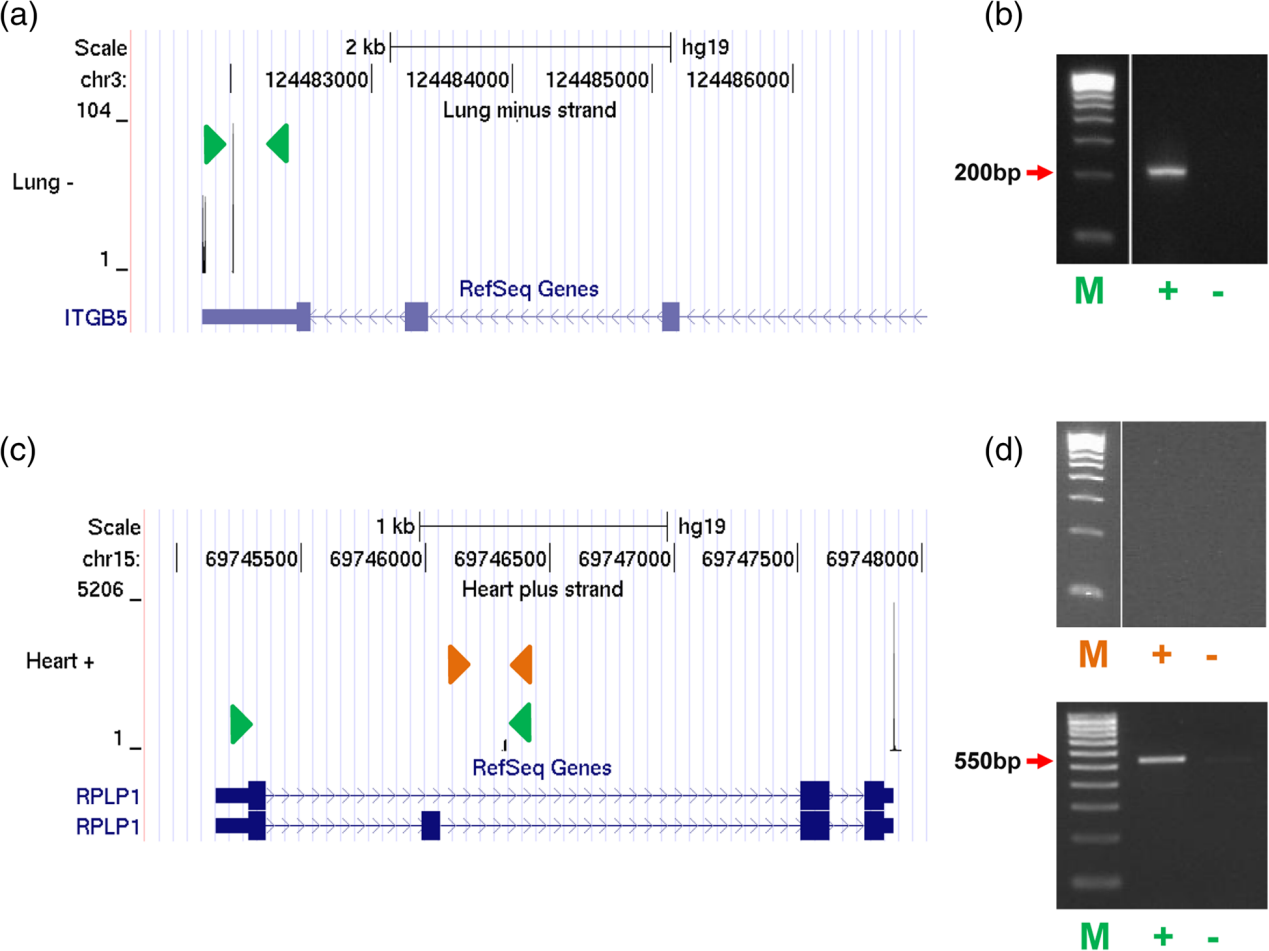
**Validation of PA clusters in 3′ UTR and intron regions. (a)** A schematic view of a novel PA site identified in the 3′ UTR region of the *ITBG5* locus. The primer pair used for validation is shown as arrowheads. **(b)** The results of RT-PCR reactions performed using an upstream primer in together with a junction primer (sequence-specific sequence + poly(T)) or a control primer (only the sequences-specific portion). The amplification product with the correct size is indicated. **(c)** Validation of a novel intronic PA site at the *PRLP1* locus. The same validation strategy was employed as before. Two primer pairs (green and orange) were designed, which use the same downstream junction primer but different upstream primers. **(d)** The RT-PCR results produced by two different primer pairs (top: orange; bottom: green). A 550bp band, which is shorter than expected size, was detected.

Since our data uncovered a large number of novel polyadenylation sites, RT-PCR was used to experimentally validate novel PA clusters located in the 3′ UTR and intronic regions (Figure 3; Additional file 8; see Methods for detail). These PA sites represent two most prevalent classes of alternative polyadenylation, and are involved in regulating 3′ UTRs length and coupling between alternative splicing and polyadenylation, respectively. All (10 out of 10) the PA clusters, which were randomly selected from the 3′ UTR regions, can be experimentally validated (Additional files 8 and 9). Similarly, 10 out of 11 novel intronic PA clusters were validated (Additional files 8 and 10). For the one case that failed the initial validation, it was due to an unannotated splicing event: the PA site was ultimately confirmed with a primer further upstream and the RT-PCR product is shorter than the expected size (Figure 3c,d).

### Sequence motifs involved in precise PA site selection

**Figure 4:**
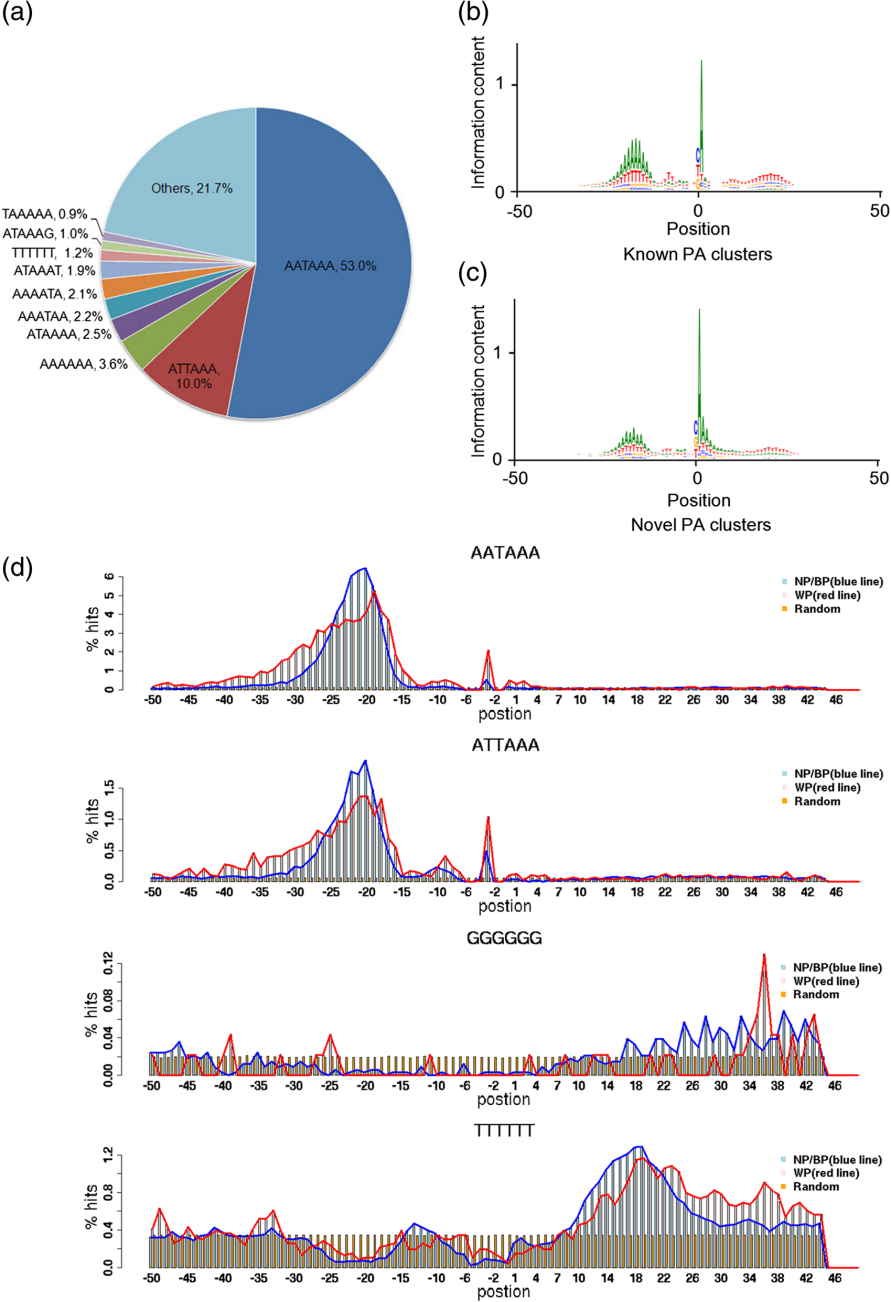
**Motif and information content analysis of all identified PA clusters. (a)** 6-mer sequence motifs enriched in the region surrounding PA clusters. The raw data from all 13 tissues were combined for PA cluster calling. The sequence from 50 nt upstream and downstream of PA clusters was extracted for motif analysis. The information contents of nucleotide sequences surrounding known and novel PA clusters are shown in **(b)** and **(c)**, respectively. The zero position is the mode of PA clusters. **(d)** Histogram of sequence motifs enriched in NP/BP and WP PA clusters. HOMER was first used to identify 6-mer motifs enriched in 100-bp regions surrounding NP/BP and WP clusters. The frequency of each enriched motif (p value < 1 × 10^-5^) is shown by evenly dividing the 100 bp into 100 bins (X-axis), with 0 denotes the mode of the cluster.

Motif analyses were then performed for the PA clusters identified above. As expected [14,23], AATAAA and ATTAAA are the top motifs overrepresented in the PA surrounding regions (Figure 4a). For both annotated and novel PA clusters, AU-rich and U/GU-rich regions are detected at upstream and downstream of PA cleavage site, respectively (Figure 4b,c), which agreed with the position of canonical poly(A) signal and downstream sequence element (DSE) in metazoans [5].

It has been shown that PA clusters may vary significantly in size and shape [19]. We therefore broadly categorized PA clusters into 3 groups: Narrow Peak (NP), Broad with Peak (BP) and Weak Peak (WP). Both NP and BP have a dominant peak (mode ± 2bp > 50% of total reads), whereas WP exhibits a more dispersed pattern of cleavage sites (see Methods for detail). Strikingly, majority of the PA clusters belong to the NP (17,183 clusters or 45.1%) and BP (16,257 clusters or 42.7%) categories. WP clusters (4,640 clusters) only account for a small percentage (12.2%) of all PA clusters. This is in sharp contrast to transcription initiation clusters where WP is the dominant class [34]. We speculated that the precision in PA site selection might be achieved by sequence conservation of *cis*-acting elements (e.g. PA signal) and/or tight regulation of polyadenylation machinery.

Next we compared the local sequence content between focused (NP and BP) and dispersed (WP) polyadenylation clusters. Interestingly, the distribution of A(A/T)TAAA motif is much tighter for the peaked (NP/BP) PA clusters than that of dispersed clusters (WP) (Figure 4d, top 2 panels), suggesting that the broad PA cleavage patterns may be resulting from multiple closely spaced polyadenylation signals. In addition, the sequences downstream of PA cleavage site differ significantly between NP/BP and WP clusters. For NP/BP clusters, they tend to have an extended G-rich region compared to the background (intergenic regions), whereas the U-rich region (‘TTTTTT’ at DNA level) is rather restricted to immediate sequences downstream of the cleavage site (Figure 4d, bottom panels). These results are consistent with notion that PA cleavage sites are often followed by U/GU-rich (DSE: downstream sequence element) and G-rich sequences (Auxiliary DSE), which are bound by CstF and CFIIm, respectively [5]. In contrast, WP clusters tend to have an extended U-rich downstream of cleave site with a narrow G-rich region at nucleotide 34–38 positions (Figure 4d). Two additional motifs, ‘UGUA’ and ‘UGUG’, were also analyzed. Consistent with the previous report [40], our results showed that for both NP/BP and WP clusters these two motifs are enriched at the upstream and downstream regions of PA cleavage sites, respectively (Additional file 11). Together, these data suggested that intrinsic sequence motifs might play an important role in determining the precision of polyadenylation cleavage events.

### Identification of PA signature in diverse tissues

**Figure 5:**
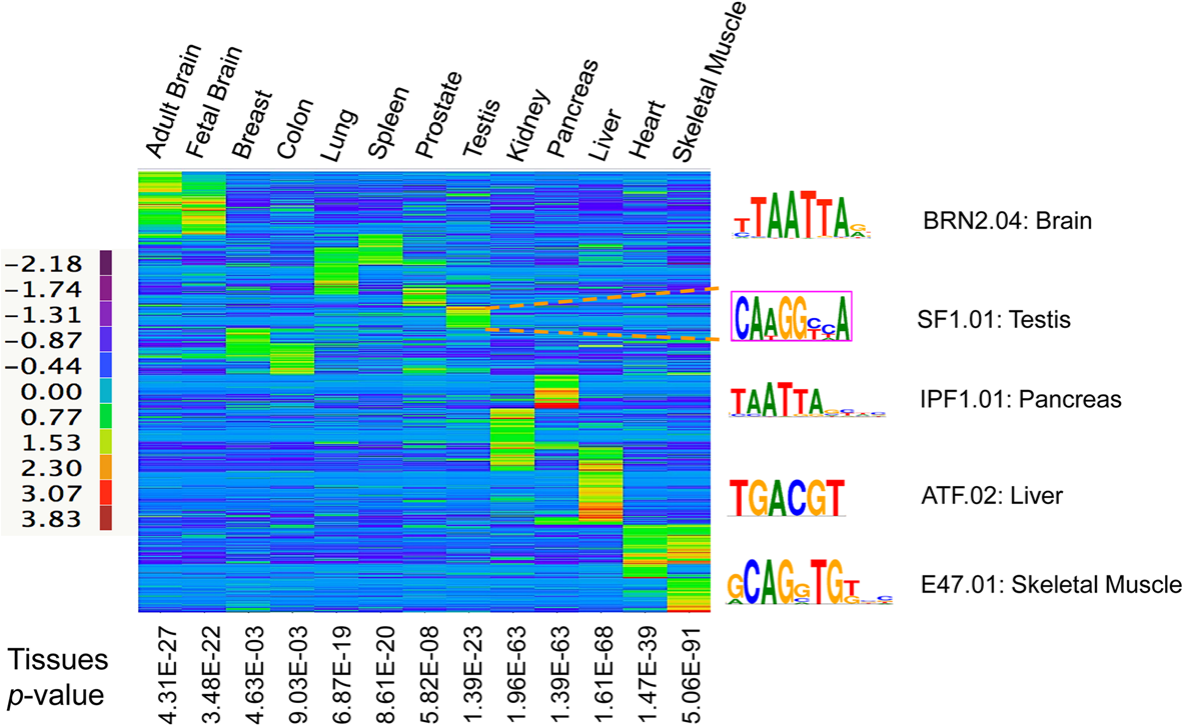
**Hierarchical clustering of identified tissue specific polyadenylation.** Two-way hierarchical clustering of 1966 tissue-enriched polyadenylation sites was performed based on Pearson correlation and the average linkage method. Normalized tag counts of individual PA clusters among 13 tissues were log2 transformed and shown as a heatmap. For each set of tissue-enriched PA clusters, tissue-specific transcriptional factor binding sites overrepresented in the promoter regions of the corresponding genes are shown on the right panel. Similarly, for genes that show tissue-restricted polyadenylation pattern, they are significantly enriched in the corresponding tissue (p values are shown at the bottom).

We next aimed to identify PA clusters that exhibit tissue-specific or tissue-enriched usage. An “entropy” term (H) was introduced to reflect PA site usage among diverse tissues [40]. For PA clusters showing low entropy, the corresponding tissue(s) with dominant PA usage were subsequently determined (Figure 5, see Methods for detail). As expected, fetal and adult brains share many tissue-specific PA clusters. Similar findings were also observed for skeletal muscle and heart. In addition, we examined whether tissue identity can be inferred from PA site usages. Starting from the host genes in which the tissue-specific PA clusters resides, the correct tissues always came out at the top with the most significant p value (9E-3 to 5E-91, Figure 5). Since the classification analysis was performed without the prior inputs on tissue origin, these results strongly suggest that PA usage, similar to gene expression profiles, also exhibits tissue-specific signatures.

Tissue specific PA sites are not necessarily resulting from tissue-specific APA. Several other mechanisms, such as tissue-specific transcription and/or alternative splicing, may lead to PA sites with preferential tissue usage. To determine the potential contribution at the transcriptional level, tissue-specific PA sites were used to compile their host transcripts, the promoter regions of which were subsequently searched for overrepresented sequence motifs. Interestingly, several transcription factor-binding sites were identified, which are known to promote tissue-specific gene expression (Figure 5, right panel). For instance, the steroidogenic factor 1 (SF1) is enriched in transcripts with testis-specific PA clusters. SF1 has recently been shown to cooperate with Sry to initiate testis development from early bipotential gonads [41]. It is worth noting that tissue-restricted expression is not the sole contributor of tissue-specific PA usage.

### 3′ UTR shortening and gene expression regulation

Alternative polyadenylation (APA) is an important mechanism for regulated eukaryotic gene expression. It has been proposed that transcriptional activity is coupled with APA [42], and highly expressed loci tend to have shortened 3′ UTR by favoring the proximal APA sites [18]. These earlier studies arbitrarily divided the 3′ UTR into “constant” and “alternative” regions based on prior APA annotations, and the shortening of 3′ UTR was inferred from microarray or RNA-seq data. Since our data contain experimentally defined PA sites as well as their counts, it provides a unique opportunity to interrogate global 3′ UTR patterning among different tissues.

**Figure 6:**
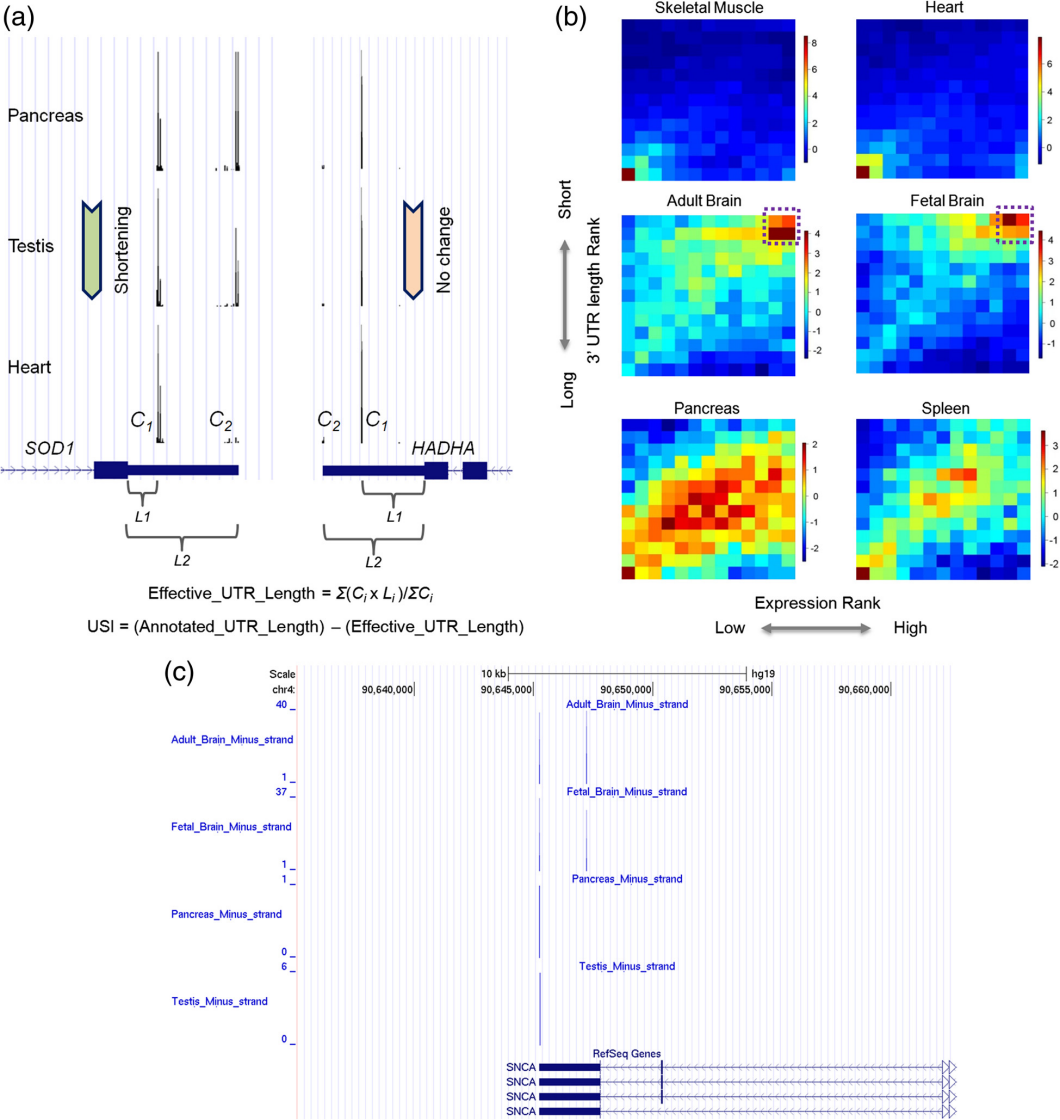
**Tissue preference of 3′ UTR shortening/lengthening. (a)** A schematic diagram for computing 3′ UTR shortening index (USI). *C*_*i*_ is the tag counts of corresponding PA cluster while *L*_*i*_ denotes the length of corresponding PA cluster. **(b)** For each given tissue, the relative expression level (X-axis) and 3′ UTR length (Y-axis) of individual genes were ranked among 13 tissues. Each box in a given 2D plot represents a specific subset of genes grouped by their ranks. The color of each box represents enrichment (Red) or depletion (Blue) of the corresponding gene group. Upper panel: two tissues (skeletal muscle and heart) enriched for genes with lower expression rank and longer 3′ UTR; Middle panel: overrepresentation of genes with higher expression rank and shorter 3′ UTR in adult and fetal brain; Bottom panel, both lowly expressed genes with longer 3′ UTR and highly expressed genes with shorter 3′ UTR are enriched in pancreas and spleen. **(c)** α-synuclein coding gene *SNCA* shows higher expression rank and shortened effective 3′ UTR in both adult and fetal brain tissues. Normalized tag number is represented in Y-axis. Known RefSeq transcripts are shown at the bottom.

To this end, we devised a UTR shortening index (USI). Based on a consolidated gene model, the USI analysis employed both the locations and the tag counts of the PA clusters identified in the 3′ UTR of each transcribed locus (Figure 6a). This is a significant improvement over the previous strategies [18,24,29], which only considered the most proximal and distal PA sites annotated for individual gene loci. In addition, the expression level of each gene was approximated by the combined counts of all PA clusters. Since protein-coding genes may differ significantly in their transcript structures (e.g. 3′ UTR length, number of PA sites), we developed a gene-centric ranking scheme for subsequent analysis. For each gene in a given tissue, its effective 3′ UTR length and normalized expression level were ranked with respect to other tissues, therefore avoiding unexpected complications due to averaging UTR lengths among genes (Figure 6b).

Three distinct enrichment patterns were discovered among tissues examined. Brain, testis, lung and breast are enriched for genes with abundant transcripts and a shortened 3′ UTR (Figure 6b middle panel and Additional file 12 middle panel), while low-abundance transcripts in several other tissues (e.g. heart, skeletal muscle) tend to have a lengthened 3′ UTR (Figure 6b top panel and Additional file 12 top panel). The rest of tissues (e.g., pancreas and spleen) are by virtue an admixture and favor both gene categories (Figure 6b bottom panel and Additional file 12 bottom panel). Despite the tissue preferences in 3′ UTR shortening/lengthening, these observations supported the notion of reverse correlation between 3′ UTR length and expression level, indicating the potential coordination between transcriptional control and alternative polyadenylation [18]. Other possibilities may also exist; for instance, 3′ UTR length might affect mRNA stability, a potential mechanism does not necessarily involve transcription regulation.

It has been proposed that 3′ UTR shortening may promote gene expression, possibly by avoiding miRNA targeting [43,44]. Conversely, transcripts with longer 3′ UTR are more likely to be targeted by miRNAs and/or other regulatory molecules. With regard to APA, two different paradigms might be involved to help establish tissue identities. The preferential usage of proximal PA sites in tissue-enriched genes (e.g. brain and testis) is expected to reinforce transcriptional decision on genes required for proper tissue functions. Supporting this notion, Gene Ontology (GO) analysis of genes with high expression rank and shortened 3′ UTR in adult and fetal brain shows significant enrichment of neural functions (Additional file 13 and Additional file 14). One interesting example is *SNCA*, which encodes α-synuclein (non A4 component of amyloid precursor) and is involved in the regulation of dopamine release and transport [45]. *SNCA* is highly expressed in adult and fetal brain compared to other tissue analyzed. In addition, it utilizes a proximal PA site only in the two brain tissues examined, while the distal PA site is employed in other tissues (e.g. pancreas and testis, Figure 6c). These data supported the notion that APA might play important roles in establishing tissue or cell identity. In contrast, certain lowly expressed transcripts might be the result of leaky transcription and 3′ UTR lengthening of these transcripts may help prevent their expression at the posttranscriptional level. It is worth noting that the two above mechanisms are not mutually exclusive. As demonstrated in the pancreas and spleen tissues (Figure 6b bottom panel), they might complement each other to ensure a broader genome-wide coordination.

## Discussion

To profile global polyadenylation events, several sequencing based methods have been developed [19,25,28,30]. In these methods, the final sequencing libraries contained a stretch of repetitive sequence introduced by oligo(dT) priming. Due to low sequence complexity downstream of the PA cleavage sites of interest, such experimental design often led to unnecessary challenges at the sequencing step (requiring long sequence read if sequenced from 5′ end or using oligo(dT) as sequencing primer, which might not be optimal, if sequenced from 3′ end). To solve this problem, our PA-seq strategy introduced a modified base (dUTP) in the oligo(dT), which can help remove the stretch of ‘T’ by Uracil-Specific Excision Reagent (USER) after second strand synthesis, and thus enables direct interrogation of polyadenylation sites in a more effective manner. Although our PA libraries can be sequenced directly from the 3′ end, paired-end sequencing was employed in this study, which is expected to further improve mapping specificity and efficiency as well as recapitulate local sequence complexity (e.g. splicing events in the 3′ UTR region). A detailed comparison between the PolyA-seq [27] and PA-seq (this study) in mappability and identification of PA sites can be found in Additional file 15 and Additional file 16, respectively.

Another innovative polyadenylation method, 3P-Seq, has also been developed [29]. One major advantage of this method is the use of splint oligo ligation to avoid internal priming events. In addition, it employed RNase H digestion to shorten poly(A) tail of the target mRNAs. However, the PA profiles resulting from 3P-Seq strategy is not as quantitative as conventional methods, possibly due to the involvement of multiple ligation steps that may not be highly efficient [27]. An alternative approach, 3′READS, has also been developed to address the internal priming issue [46]. These methods can be used to generate a reference list of authentic PA sites, which can help further improve the computational strategy of our PA-seq analysis to achieve more reliable and quantitative profiling of genome-wide polyadenylation events.

Alternative polyadenylation is known to be an important mechanism in regulating tissue-specific gene expression and proteome diversity. One striking observation from this study is the distinct tissue preference in 3′ UTR shortening/lengthening. Since our analyses focused on tandem PA sites in the 3′ UTR region, it is unlikely that 3′ UTR shortening/lengthening is a direct result of alternative splicing. However, it does not rule out that splicing factors, in addition to other RNA binding proteins, may play a direct role in controlling PA choices. An early profiling study has identified the expression signature of splicing factors in different tissues; and snRNP (small nuclear ribonucleoprotein particle) is among the splicing factor genes that are highly differentially expressed particular in brain and testis [47]. Accumulated evidence showed that U1 snRNP plays a determinative role in regulating polyadenylation and mRNA length [24,48]. Therefore, it will be of great interest to further characterize the direct involvement of component of basal and alternative splicing machinery in regulated polyadenylation.

Strikingly, we found selective 3′ UTR shortening in genes highly expressed in fetal and adult brain, while others have found that brain transcripts on average have the longest 3′ UTR in length [32], a subset of which may even have extended 3′ UTR beyond current annotations [49]. Instead of averaging among transcribed loci, our analysis is a gene-centric approach, comparing the normalized expression level as well as the effective UTR length of the same gene among diverse tissues, to uncover potential coordination between gene expression and polyadenylation. These opposing observations may not be contradictory as they seem to be at the initial glimpse. Together, these data implied that a subset of brain-enriched genes preferentially shorten their 3′ UTRs to ensure a high level of expression, despite that a different subset of transcripts expressed in the brain may have a rather extended 3′ UTR. As one prevalent phenomenon in neurons, it has been shown that brain-enriched miRNAs tend to co-express with their target genes, which also exhibit brain-specific expression [50]. The paradox can be in part explained by selective 3′ UTR shortening and target avoidance, suggesting alternative polyadenylation might be critical for establishing neuronal cell identity.

Lastly, we found ncRNA, similar to protein coding transcripts, are also extensively regulated at the polyadenylation level. The data sets presented here provided a starting point to further characterize the functional significance of such regulatory events. It is plausible that different ncRNA isoforms may have distinct regulatory functions, cellular localization and/or stability. For instance, comparison of polyadenylation transcripts at different cellular compartments is expected to interrogate potential links between alternative polyadenylation and subcellular localization. Further integration of function and interaction data (e.g. the secondary structure of noncoding transcripts with or without alternative region) is required for to characterizing this additional level of transcriptome complexity and deserves future study.

## Conclusions

One major challenge of profiling transcriptome 3′ end is to avoid sequencing the poly(A) stretch. We used Uracil-Specific Excision Reagent to recognize and cleave the modified base (dUTP) near the polyadenylation site, therefore eliminating the potential complication due to these low complexity sequences. Using this modified PA-seq strategy, tissue-specific PA signatures were identified. Furthermore, we showed that noncoding transcripts, similar to protein-coding genes, are extensively regulated at the 3′ end. In addition, downstream G-rich motif and upstream U-rich motif played an important role in the regulation of tissue-specific polyadenylation. Lastly, we showed distinct patterns of 3′ UTR shortening/lengthening among different tissues, which suggested that APA may play a critical role in establishing tissue or cell identity. Together, the PA-seq strategy provided a comprehensive polyadenylation landscape of human transcripts across diverse tissues.

## Methods

### PA-seq library construction

Total RNA derived from 13 normal human tissues, including adult brain, fetal brain, kidney, liver, prostate, testis, breast, colon, heart, lung, pancreas, spleen and skeletal muscle were purchased from Biochain or Clontech. 10 μg of DNA-free total RNA was sheared into 200–300 nt fragments by heating (94°C for 3 minutes) with magnesium. Sheared RNA was precipitated by ethanol with GlycoBlue (Ambion) as a carrier. Reverse transcription was carried out using a modified oligo(dT) primer (5′-bio-TTTTTTTTTTTTTTTTdUTTTVN-3′, ‘bio’ denotes duo biotin group, ‘dU’ stands for deoxyuricile, ‘V’ represents any nucleotide except T and ‘N’ denotes any nucleotide). Incubate the reverse transcription reaction at 42°C for 2 min before adding Superscript reverse transcriptase II (Invitrogen) to increase specificity. After second strand synthesis, Dynabeads MyOne C1 (Invitrogen) was used to pull down the resulting dsDNA. Incubate the beads with APex Heat-Labile Alkaline Phosphatase (Epicentre) to remove phosphate group, which enables strand specificity at the later PCR step since only the bottom strand cDNA can be ligated and thus amplified. To release dsDNA from MyOne beads, USER enzyme (NEB) was added. The released dsDNA was then end repaired, followed by adding an ‘A’ base at the ends. Illumina paired-end Y linker was ligated and size selected. A 16-cycle PCR was then carried out with Phusion Hot Start High-Fidelity DNA Polymerase (Finnzymes) to generate the final PA-seq libraries, which were sequenced using Illumina HiSeq2000 platform.

### Paired-end mapping

Paired-end reads were split into 13 tissue samples by barcode and further correct the strand according to ‘TTT’ at the beginning of the reads. Processed raw data were then aligned by bwa [51] to human genome (version hg19) allowing two mismatches and processed by samtools [52]. All uniquely mapped pairs were used for downstream analyses.

### Comparison of PA-seq data with PolyA_DB and RefSeq

PolyA_DB (version 1, only contain human and mouse poly(A) sites) was downloaded from Bin Tian’s website (http://exon.umdnj.edu/polya_db/) [14]. PolyA_DB data was collected from all cDNA/ESTs sequences in the UniGene database from NCBI (July and August 2005 version) and aligned to genome sequences build hg17 using BLAT. ESTs sequences came from a variety of tissues including eye, retina, skin, testis, pancreas, stomach, colon, brain and mixed tissues. For PolyA_DB sequences, coding and intronic regions were taken into consideration for those falling in 3′ UTR with 1kb upstream and 1kb downstream. For PA-seq data, we selected those pairs with more than one unique 5′ reads. Since in PolyA_DB, each gene has at least one poly(A) site, some genes actually appear more than once, and hence the minimum distance varies accordingly. For comparison with RefSeq annotation, we download the database from UCSC. Both protein-coding and non-coding genes were computed. All mappable pairs and non-redundant pairs were calculated separately.

### Peak calling and cluster analysis

After mapping, the information of exact cleavage and polyadenylation sites was extracted. F-seq [35] was used for the peak calling of PA sites. The PA-seq data from 13 tissues were combined so that a unified peak-calling scheme can be applied. We resized the PA clusters to the shortest distance that contained 95% of the reads according to our previous publication [34]. To filter internal priming, we removed PA clusters with 15 ‘A’ in the 20 nucleotides region downstream of peak mode. With a minimum of 50 tags, we classified the PA clusters into three patterns by the following definitions [34]: Narrow Peak (NP) clusters contained ≥ 50% of the reads within ±2 nt of the mode and span < 10 nt; Broad with Peak (BP) clusters were those that contained ≥ 50% of the reads within ±2 nt of the mode and are ≥ 10 nt in length; All other clusters were classified as Weak Peak (WP). RefSeq annotation was used for the genomic location analysis. For peaks falling into multiple categories, we set priority as PA > 3′-UTR > Extended_3′-UTR > Exon > Intron > 5′-UTR > TSS > Promoter. PA defined as annotated PA site plus upstream and downstream 10bp. Extended_3′-UTR denotes downstream 1 kb of 3′ UTR. TSS represents exact transcriptional start site plus upstream and downstream 10bp. Promoter defined as upstream 250 bp of TSS. For Figure 4d, we applied 5 bp window as threshold of NP and BP.

### Motif and information content analysis

Homer software from UCSD (http://biowhat.ucsd.edu/homer/) was used to perform the motif analysis in Figure 4d and Additional file 11. 6mer motifs were analyzed. Weblogo version 3 (http://weblogo.threeplusone.com) was used to draw the information content with classic color scheme for Figure 4b,c. Top four enriched 6mer motifs (p value < 1 × 10^-5^) were counted in 100-bp regions surrounding NP/BP and WP PA clusters separately. 4000 random intergenic sequences each with 100 bp in length were also scanned by top four enriched 6mer motifs. The frequency of each enriched motif was calculated by normalizing total input sequences.

### Shannon entropy analysis of tissue-specific polyadenylation

Shannon entropy [40] was used for the analysis of identifying tissue-specific polyadenylation sites. First, we normalized each tissue PA-seq data by its library size, and then performed quantile normalization for all 13 tissues PA-seq data. Shannon entropy score H was computed for each polyadenylation site. Q scores were computed based on H score to estimate the expression specificity of each PA site for a particular tissue. Based on H and Q scores’ distribution and variation, we applied the cut-off corresponding approximately less than the median minus two standard deviations to identify the regulated tissue specific polyadenylation sites. On the contrary, we detected constitutive PA sites when both H and Q values are greater than mean plus two standard deviations.

### Clustering of tissue specific gene expression

Unsupervised two-way hierarchical clustering of 13 tissues and identified tissue specific polyadenylation sites was performed based on Pearson correlation and the average linkage method. In each specific tissue cluster, 1kb upstream sequences from PA cluster start site were scanned for finding transcription factor (TF) binding sites common to at least 60% of input sequences by MatInspector in Genomatix Genome Analyzer. Tissue-specific TFs were selected by p value < 10^-7^.

### Gene ontology analysis

DAVID (Database for Annotation, Visualization and Integrated Discovery) [53] was used for the GO analysis of enriched genes from Figure 6b. Three terms (GOTERM_BP_FAT, GOTERM_CC_FAT and GOTERM_MF_FAT) were selected for analysis. The top ten GO terms in the “Functional Annotation Chart” were shown in Additional file 13 and Additional file 14. The default population background in enrichment calculation consists of all corresponding genes in the genome that have at least one annotation in the analyzing categories.

### Define 3′ UTR shortening index

We used Entrez gene ID to cluster the RefSeq transcript IDs. Overlapping genes on the same strand were removed from further consideration to avoid wrongful assignment of PA site to 3′ UTR. Furthermore, we restricted our attention to genes (represented by Entrez IDs) whose annotated transcripts (represented by RefSeq IDs) that shared the same stop codon. Based on the consolidated gene model above, the pipeline considers both the locations *(L)* and the tag counts *(C)* of the PA clusters identified in the 3′ UTR of each transcribed locus to compute “Effective_UTR_Length” (Effective_UTR_Length = *Σ*(*C*_*i*_ × *L*_*i*_)/*Σ*C_*i*_, see Figure 6a). 3′ UTR shortening index (USI) is defined as the length difference between annotated and effective 3′ UTR (USI = Annotated_UTR_Length − Effective_UTR_Length, see Figure 6a). The effective 3′ UTR length and PA cluster expression were ranked from low to high separately among 13 tissues. Each box in a given 2D plot (Figure 6b) represents Z-score of the observed number of PA clusters (genes) in the specific tissue.

### RT-PCR validation of PA clusters

To validate the mode of a PA cluster, a junction primer (+, half complementary to 3′ end sequence and half complementary to poly(A) sequence) together with an upstream gene-specific primer were designed by Primer3 (version 0.4.0) and synthesized by IDT. The control primer (−) lacks the sequence complementary to poly(A). Reverse transcription of 1μg DNA-free total RNA was performed with SuperScript II reverse transcriptase (Invitrogen) in a 20 μl reaction, containing 4 pmol oligo(dT) primer (5′-TTTTTTTTTTTTTTTTTTTTVN-3′), 40 units of RNasin (Promega) and 6 ng/μl freshly-made actinomycin D. RT reaction was incubated at 42°C for 2 min before adding reverse transcriptase. We then incubate the reaction at 42°C for 60 min and 75°C for 15 min. PCR was performed in a 20 μl reaction, containing 1 μl of RT template, 1× PCR buffer (Qiagen), 0.4 nmol dNTP, 0.4 pmol of each forward and reverse primer, and 1 μl of Taq DNA Polymerase (Qiagen). Thermal cycling was carried out as the following: 94°C for 30s; 30 ~ 40 cycles of 94°C for 30s, 52 ~ 58°C for 30s and 72°C for 30s; 72°C for 10 min; hold at 4°C. 3μl of PCR product was taken out and run in a 2% agarose gel.

### Data access

PA-seq raw data can be found at the NCBI Sequence Read Archive (SRA) with submission number SRA059064. Custom tracks of 13 tissues can be found in Additional file 17.

### Additional information

A step-by-step protocol of PA-seq can be found in Additional file 18.

## Competing interests

The authors declare that they have no competing interests.

## Authors’ contributions

TN and JZ developed the library construction protocol. KK contributed to the experimental validation. TN and YW carried out the deep sequencing. YY, DH, WY, UO and WP contributed to the data analyses. TN and JZ wrote the manuscript. All authors read and approved the final manuscript.

## Supplementary Material

Additional file 1 Mapping summary of PA-seq paired-end reads. Click here for file

Additional file 2 **Distribution of all PA-seq 3′ reads relative to annotated RefSeq poly(A) sites.** For genes with multiple annotated PA sites in the RefSeq database, only the longest poly(A) site is included. Click here for file

Additional file 3 **Distribution of non-redundant PA-seq 3′ reads relative to annotated RefSeq poly(A) sites.** For genes with multiple annotated PA sites in RefSeq database, only the longest poly(A) site is included. Click here for file

Additional file 4 **Expression correlation between Affymetrix array and PA-seq data.** Log2 transformed values of Affymetrix array (X-axis) and PA-seq reads (Y-axis) in liver are shown. R denotes correlation coefficient. For PA-seq, total mappable reads were used for expression comparison. Click here for file

Additional file 5 **Expression correlation between PA-seq and RNA-seq data.** Additional RNA-seq data was obtained for the same human kidney sample used in the PA-seq analysis. We used total mappable reads for expression comparison. The expression levels of individual gens were computed based on RPKM (reads per kb per million) and RPM (reads per million) for RNA-seq and PA-seq, respectively. a) The expression correlation between RNA-seq (X-axis) and PA-seq (Y-axis) is shown as a scatter plot, and all mappable reads in the PA-seq were included in the analysis. b) is the same as a) except that PA-seq were further processed to remove sequence reads that are not in the PA clusters identified. Click here for file

Additional file 6 **Distribution of 7714 distant PA clusters.** Distant PA clusters were categorized into 3 groups, NP, BP and WP, using the same criteria as genic PA clusters. Briefly, NP (narrow peak) clusters were defined as ≥ 50% of the reads within ±2 nt of the mode and the cluster size is < 10 nt; BP (Broad with Peak) clusters were those clusters that contain ≥ 50% of the reads within ±2 nt of the mode and are ≥ 10 nt in length; All other clusters were classified as Weak Peak (WP). (see Methods for detail). The number as well as the percentage of distant PA clusters in NP, BP and WP categories are shown. Click here for file

Additional file 7 **The usage of PA sites among human tissues.** As a stringent cutoff, a PA cluster (or PA site) is defined as “utilized in a tissue” only if its tag count is greater than the median tag count (quantile normalized) of the corresponding tissue. a) The usage of all PA clusters identified by PA-seq among 13 tissues. For each PA cluster, we first determined the number of tissues it is utilized, and the overall distribution of PA usages is shown in a histogram. In summary, majority (~87%) of the PA clusters can be found in 5 or more tissues (y-axis) with 35.53% of the clusters were detected in all 13 tissues. In addition, 13.36% of all PA clusters showed a relatively tissue-restricted usage in less than 5 tissues. b) The usage of 7714 distant PA clusters among 13 tissues. The same analysis was performed as a). Overall, ~90% of the distant PA clusters were employed in 5 or more tissues (y axis), and 17.92% of the clusters can be detected in all 13 tissues. 10.11% PA clusters showed a relatively tissue-restricted usage in less than 5 tissues. Click here for file

Additional file 8 **RT-PCR validation of novel PA clusters located in intronic or intergenic regions.** a) A schematic diagram for experimental validation of PA clusters. A junction primer (+, half complementary to the region immediately upstream of PA cleavage site and half complementary to polyA sequence), in together with an upstream gene-specific primer, was used to validate the corresponding polyA site. A control primer (−), which contains only the gene-specific portion of the junction primer, served as a negative control. For an authentic polyA site, the junction primer is expected to produce a specific band, but the control primer fails to do so because of its lower melting temperature (Tm). Novel poly(A) sites in 3′ UTR (b) and intronic (c) region were validated by a junction primer (+, half complementary to 3′ end sequence and half complementary to polyA sequence) together with an upstream primer. The control primer (−) lacks the sequence complementary to poly(A). Click here for file

Additional file 9 Primer pairs for validation of PA clusters in 3′ UTR. Click here for file

Additional file 10 Primer pairs for validation of PA clusters in intronic regions. Click here for file

Additional file 11 **Enrichment of UGUA and UGUG motifs in NP/BP and WP PA clusters.** Two 4-mer motifs, UGUA and UGUG (TGTA and TGTG at the DNA level were scanned in 100-bp regions surrounding NP/BP and WP clusters. We evenly divided the 100 bp regions into 20 bins (X-axis) and the motif frequencies (Y-axis) of individual bins were plotted. The “0” position denotes the mode of the PA cluster. Click here for file

Additional file 12 **Distinct patterns of 3′ UTR shortening/lengthening in human tissues.** Upper panel, genes with lower expression rank and longer 3′ UTR are enriched in specified tissue (Kidney and Liver); Middle panel, genes with higher expression rank and shorter 3′ UTR are enriched in specified tissue (Testis, Lung and Breast); Bottom panel, both lowly expressed genes with longer 3′ UTR and highly expressed genes with shorter 3′ UTR are enriched in specified tissue (Pancreas and Colon). X-axis reflects the expression ranking among 13 tissues for each gene. Y-axis denotes the ranking of average 3′ UTR length. Color bar shows the enrichment of genes in each tissue. Red denotes enrichment while blue represents depletion. Click here for file

Additional file 13 GO analysis of genes (top right corner in dashed square) in Adult Brain. Click here for file

Additional file 14 GO analysis of genes (top right corner in dashed square) in Fetal Brain. Click here for file

Additional file 15 Mapping quality comparison between our data and data published by Derti et al. Click here for file

Additional file 16 Comparison of PA peaks identified by PolyA-seq (Derti) and PA-seq (this study). Click here for file

Additional file 17 Custom tracks (wiggle format) of PA-seq for 13 human tissues. Click here for file

Additional file 18 A step-by-step protocol of PA-seq. Click here for file
