# Supplementary material for: Distinct polyadenylation landscapes of diverse human tissues revealed by a modified PA-seq strategy
Source: BMC Genomics. 2013 Sep 11;14:615. doi: 10.1186/1471-2164-14-615 (PMC3848854; doi:10.1186/1471-2164-14-615)
Supplement: Additional file 1 — Mapping summary of PA-seq paired-end reads. [file 1471-2164-14-615-S1.pdf]

Additional file 1. Mapping summary of PA-seq paired-end reads

Tissue	Raw pairs*	Mapped pairs**	% of Mapped pairs	Uniquely mapped pairs	% of Uniquely mapped pairs
Adult_Brain	8,210,666	5,954,284	73%	5,530,978	67%
Fetal_Brain	9,896,201	7,448,859	75%	6,894,447	70%
Breast	14,880,212	12,892,188	87%	11,510,671	77%
Colon	7,834,482	6,860,861	88%	6,169,808	79%
Heart	14,977,686	13,649,087	91%	12,946,911	86%
Kidney	8,049,781	7,244,408	90%	6,857,322	85%
Liver	2,853,453	2,460,764	86%	2,313,254	81%
Lung	9,403,484	8,412,258	89%	7,656,966	81%
Pancreas	7,541,189	5,551,627	74%	4,979,939	66%
Prostate	4,675,004	4,246,285	91%	3,872,630	83%
Skeletal muscle	5,012,646	4,236,455	85%	4,027,817	80%
Spleen	6,766,253	5,912,111	87%	5,144,279	76%
Testis	7,558,075	6,855,270	91%	6,308,689	83%
Total	107,659,132	91,724,457	85%	84,213,711	78%

*Raw pairs mean after barcode split and removal of reads with TTT in both read1 and read2.

**bwa aligner allowing two mismatches was used for paired-end mapping.
