# Supplementary material for: Distinct polyadenylation landscapes of diverse human tissues revealed by a modified PA-seq strategy
Source: BMC Genomics. 2013 Sep 11;14:615. doi: 10.1186/1471-2164-14-615 (PMC3848854; doi:10.1186/1471-2164-14-615)

Additional file 2. Distribution of all PA-seq 3' reads relative to annotated RefSeq poly(A) sites.

For genes with multiple annotated PA sites in the RefSeq database, only the longest poly(A) site is included.

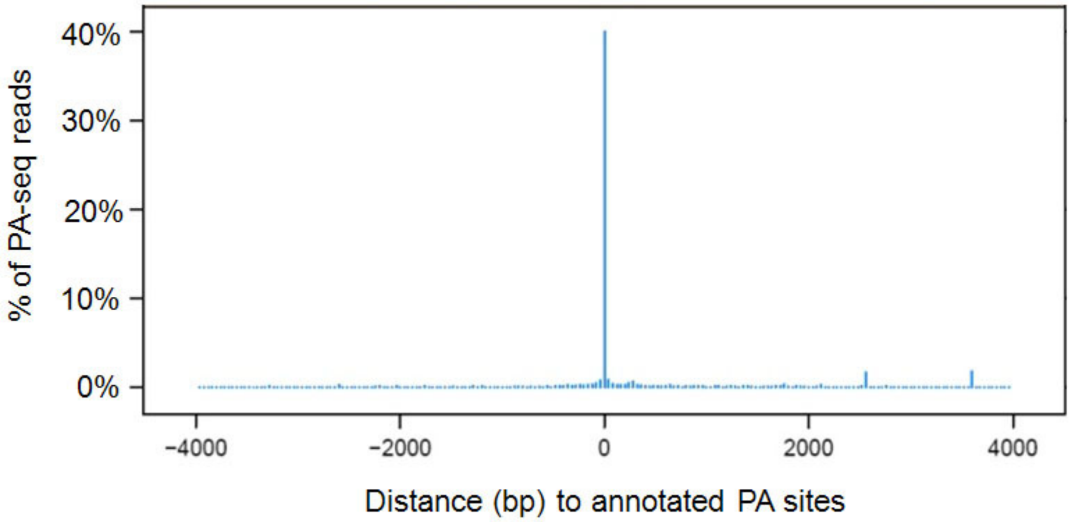

Supplement: Additional file 2 — Distribution of all PA-seq 3′ reads relative to annotated RefSeq poly(A) sites. For genes with multiple annotated PA sites in the RefSeq database, only the longest poly(A) site is included. [file 1471-2164-14-615-S2.pdf]
