# Supplementary material for: Distinct polyadenylation landscapes of diverse human tissues revealed by a modified PA-seq strategy
Source: BMC Genomics. 2013 Sep 11;14:615. doi: 10.1186/1471-2164-14-615 (PMC3848854; doi:10.1186/1471-2164-14-615)

Additional file 4. Expression correlation between Affymetrix array and PA-seq data. Log2 transformed values of Affymetrix array (X axis) and PA-seq reads (Y axis) in liver are shown. R denotes correlation coefficient. For PA-seq, total mappable reads were used for expression comparison.

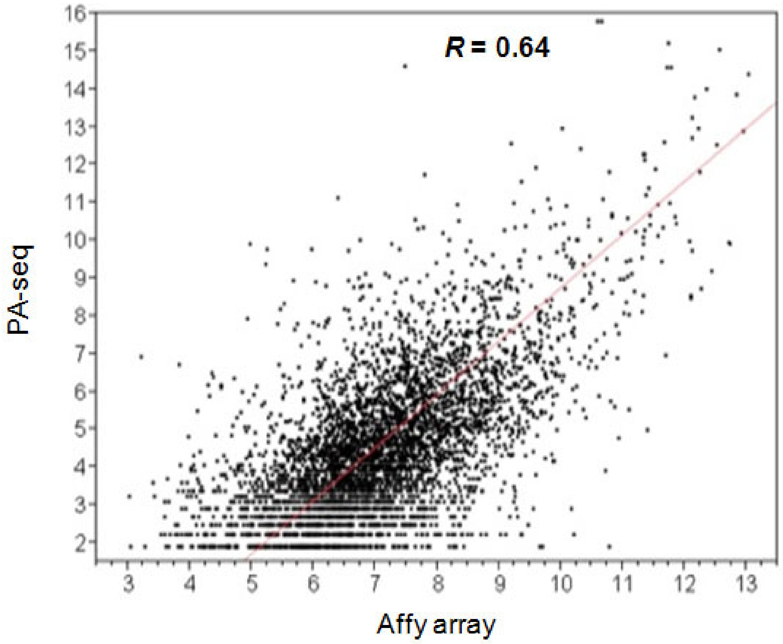

Supplement: Additional file 4 — Expression correlation between Affymetrix array and PA-seq data. Log2 transformed values of Affymetrix array (X-axis) and PA-seq reads (Y-axis) in liver are shown. R denotes correlation coefficient. For PA-seq, total mappable reads were used for expression comparison. [file 1471-2164-14-615-S4.pdf]
