# Supplementary material for: Distinct polyadenylation landscapes of diverse human tissues revealed by a modified PA-seq strategy
Source: BMC Genomics. 2013 Sep 11;14:615. doi: 10.1186/1471-2164-14-615 (PMC3848854; doi:10.1186/1471-2164-14-615)

Additional file 5. Expression correlation between PA-seq and RNA-seq data.

Additional RNA-seq data was obtained for the same human kidney sample used in the PA-seq analysis. We used total mappable reads for expression comparison. The expression levels of individual gens were computed based on RPKM (reads per kb per million) and RPM (reads per million) for RNA-seq and PA-seq, respectively. a) The expression correlation between RNA-seq (x-axis) and PA-seq (y-axis) is shown as a scatter plot, and all mappable reads in the PA-seq were included in the analysis. b) is the same as a) except that PA-seq were further processed to remove sequence reads that are not in the PA clusters identified.

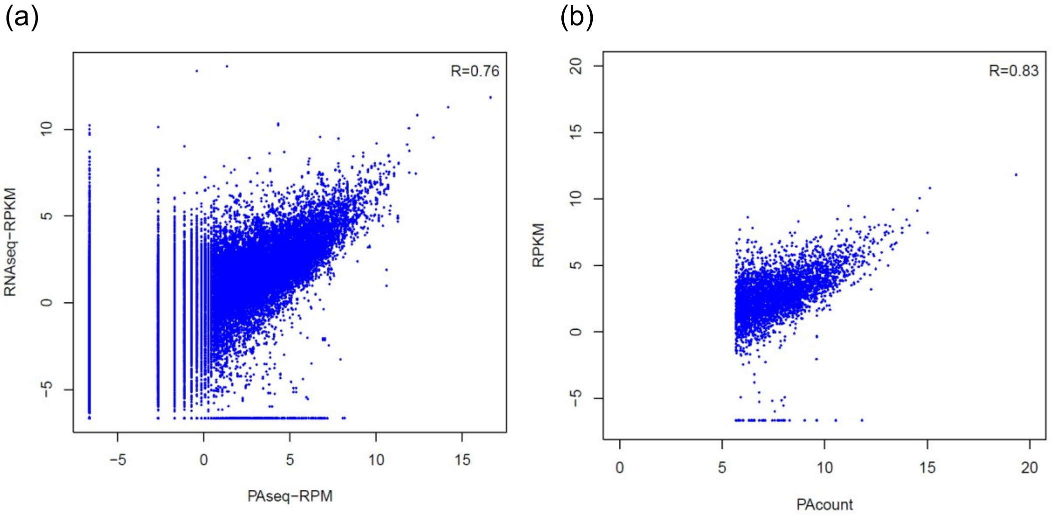

Supplement: Additional file 5 — Expression correlation between PA-seq and RNA-seq data. Additional RNA-seq data was obtained for the same human kidney sample used in the PA-seq analysis. We used total mappable reads for expression comparison. The expression levels of individual gens were computed based on RPKM (reads per kb per million) and RPM (reads per million) for RNA-seq and PA-seq, respectively. a) The expression correlation between RNA-seq (X-axis) and PA-seq (Y-axis) is shown as a scatter plot, and all mappable reads in the PA-seq were included in the analysis. b) is the same as a) except that PA-seq were further processed to remove sequence reads that are not in the PA clusters identified. [file 1471-2164-14-615-S5.pdf]
