# Supplementary material for: Distinct polyadenylation landscapes of diverse human tissues revealed by a modified PA-seq strategy
Source: BMC Genomics. 2013 Sep 11;14:615. doi: 10.1186/1471-2164-14-615 (PMC3848854; doi:10.1186/1471-2164-14-615)

Additional file 6. Distribution of 7714 distant PA clusters.

Distant PA clusters were categorized into 3 groups, NP, BP and WP, using the same criteria as genic PA clusters. Briefly, NP (narrow peak) clusters were defined as ≥ 50% of the reads within ±2 nt of the mode and the cluster size is < 10 nt; BP (Broad with Peak) clusters were those clusters that contain ≥ 50% of the reads within ±2 nt of the mode and are ≥ 10 nt in length; All other clusters were classified as Weak Peak (WP). (see Methods for detail). The number as well as the percentage of distant PA clusters in NP, BP and WP categories are shown.

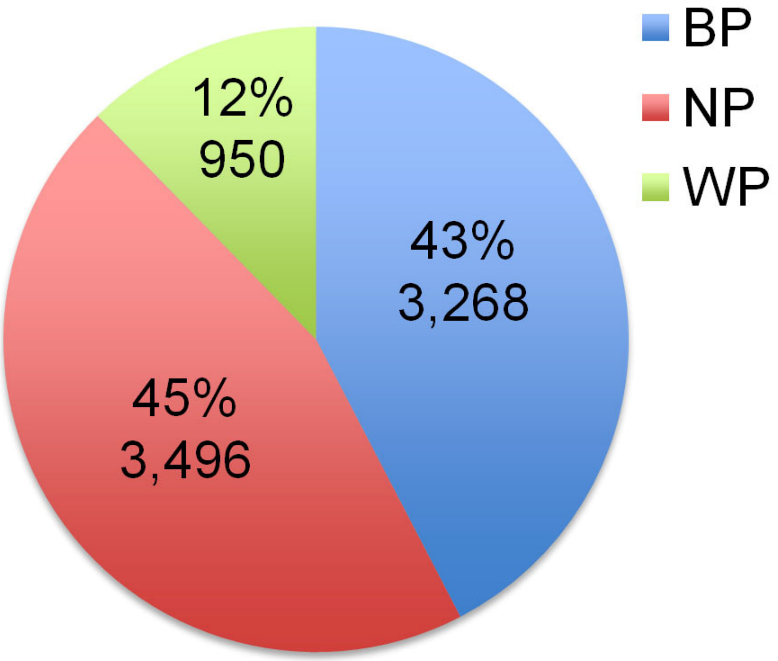

Supplement: Additional file 6 — Distribution of 7714 distant PA clusters. Distant PA clusters were categorized into 3 groups, NP, BP and WP, using the same criteria as genic PA clusters. Briefly, NP (narrow peak) clusters were defined as ≥ 50% of the reads within ±2 nt of the mode and the cluster size is < 10 nt; BP (Broad with Peak) clusters were those clusters that contain ≥ 50% of the reads within ±2 nt of the mode and are ≥ 10 nt in length; All other clusters were classified as Weak Peak (WP). (see Methods for detail). The number as well as the percentage of distant PA clusters in NP, BP and WP categories are shown. [file 1471-2164-14-615-S6.pdf]
