# Supplementary material for: Distinct polyadenylation landscapes of diverse human tissues revealed by a modified PA-seq strategy
Source: BMC Genomics. 2013 Sep 11;14:615. doi: 10.1186/1471-2164-14-615 (PMC3848854; doi:10.1186/1471-2164-14-615)
Supplement: Additional file 7 — The usage of PA sites among human tissues. As a stringent cutoff, a PA cluster (or PA site) is defined as “utilized in a tissue” only if its tag count is greater than the median tag count (quantile normalized) of the corresponding tissue. a) The usage of all PA clusters identified by PA-seq among 13 tissues. For each PA cluster, we first determined the number of tissues it is utilized, and the overall distribution of PA usages is shown in a histogram. In summary, majority (~87%) of the PA clusters can be found in 5 or more tissues (y-axis) with 35.53% of the clusters were detected in all 13 tissues. In addition, 13.36% of all PA clusters showed a relatively tissue-restricted usage in less than 5 tissues. b) The usage of 7714 distant PA clusters among 13 tissues. The same analysis was performed as a). Overall, ~90% of the distant PA clusters were employed in 5 or more tissues (y axis), and 17.92% of the clusters can be detected in all 13 tissues. 10.11% PA clusters showed a relatively tissue-restricted usage in less than 5 tissues. [file 1471-2164-14-615-S7.pdf]

Additional file 7. The usage of PA sites among human tissues.

As a stringent cutoff, a PA cluster (or PA site) is defined as "utilized in a tissue" only if its tag count is greater than the median tag count (quantile normalized) of the corresponding tissue.

a) The usage of all PA clusters identified by PA-seq among 13 tissues. For each PA cluster, we first determined the number of tissues it is utilized, and the overall distribution of PA usages is shown in a histogram. In summary, majority (~87%) of the PA clusters can be found in 5 or more tissues (y-axis) with 35.53% of the clusters were detected in all 13 tissues. In addition, 13.36% of all PA clusters showed a relatively tissue-restricted usage in less than 5 tissues.

b) The usage of 7714 distant PA clusters among 13 tissues. The same analysis was performed as a). Overall, ~90% of the distant PA clusters were employed in 5 or more tissues (y axis), and 17.92% of the clusters can be detected in all 13 tissues. 10.11% PA clusters showed a relatively tissue-restricted usage in less than 5 tissues.

(a)

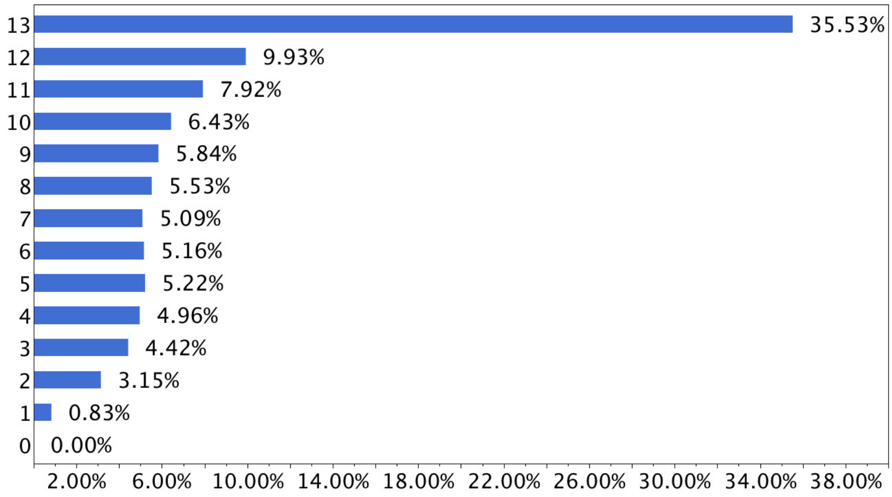

% of total (sum_qnorm > Q50)

(b)

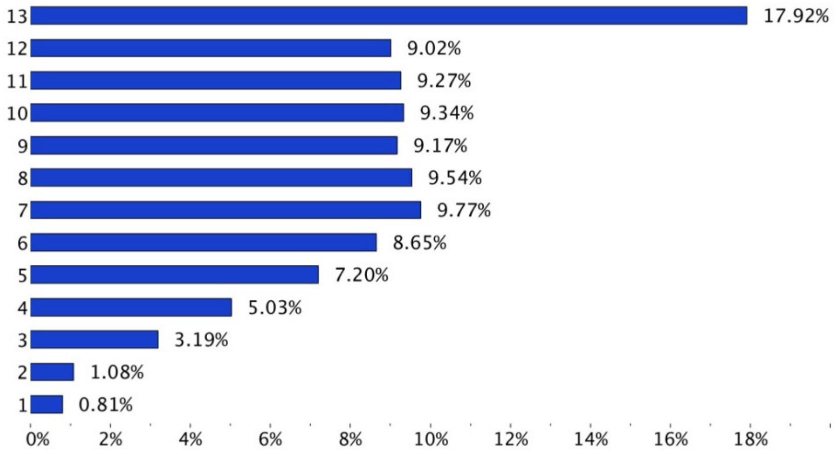

% of total (sum_qnorm > Q50)
