# Supplementary material for: Distinct polyadenylation landscapes of diverse human tissues revealed by a modified PA-seq strategy
Source: BMC Genomics. 2013 Sep 11;14:615. doi: 10.1186/1471-2164-14-615 (PMC3848854; doi:10.1186/1471-2164-14-615)

Additional file 8. RT-PCR validation of novel PA clusters located in intronic or intergenic regions.

a) A schematic diagram for experimental validation of PA clusters. A junction primer (+, half complementary to the region immediately upstream of PA cleavage site and half complementary to polyA sequence), in together with an upstream gene-specific primer, was used to validate the corresponding polyA site. A control primer (-), which contains only the gene-specific portion of the junction primer, served as a negative control. For an authentic polyA site, the junction primer is expected to produce a specific band, but the control primer fails to do so because of its lower melting temperature (Tm). Novel poly(A) sites in 3' UTR (b) and intronic (c) region were validated by a junction primer (+, half complementary to 3' end sequence and half complementary to polyA sequence) together with an upstream primer. The control primer (-) lacks the sequence complementary to poly(A).

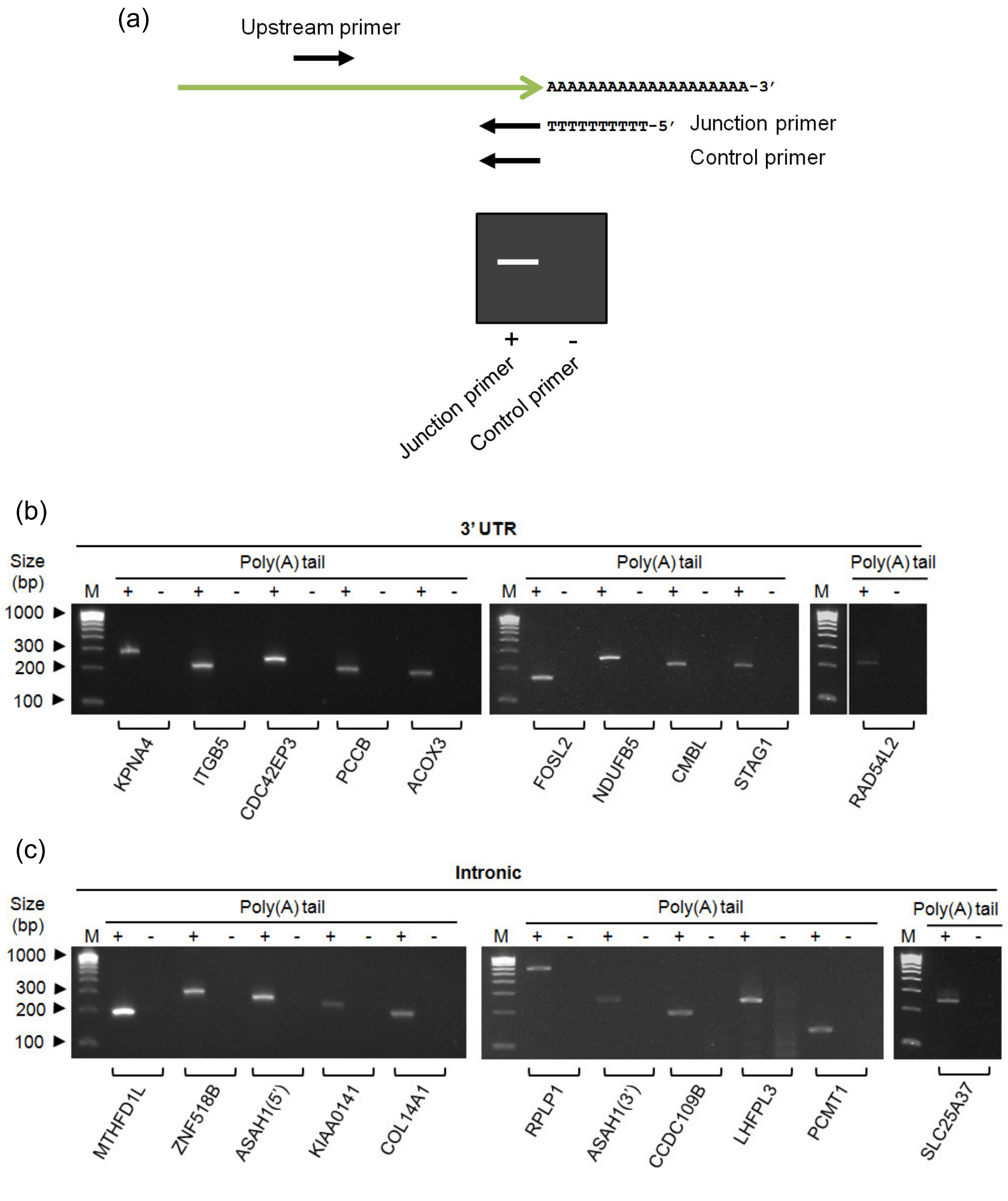

Supplement: Additional file 8 — RT-PCR validation of novel PA clusters located in intronic or intergenic regions. a) A schematic diagram for experimental validation of PA clusters. A junction primer (+, half complementary to the region immediately upstream of PA cleavage site and half complementary to polyA sequence), in together with an upstream gene-specific primer, was used to validate the corresponding polyA site. A control primer (−), which contains only the gene-specific portion of the junction primer, served as a negative control. For an authentic polyA site, the junction primer is expected to produce a specific band, but the control primer fails to do so because of its lower melting temperature (Tm). Novel poly(A) sites in 3′ UTR (b) and intronic (c) region were validated by a junction primer (+, half complementary to 3′ end sequence and half complementary to polyA sequence) together with an upstream primer. The control primer (−) lacks the sequence complementary to poly(A). [file 1471-2164-14-615-S8.pdf]
