# Supplementary material for: Distinct polyadenylation landscapes of diverse human tissues revealed by a modified PA-seq strategy
Source: BMC Genomics. 2013 Sep 11;14:615. doi: 10.1186/1471-2164-14-615 (PMC3848854; doi:10.1186/1471-2164-14-615)
Supplement: Additional file 9 — Primer pairs for validation of PA clusters in 3′ UTR. [file 1471-2164-14-615-S9.pdf]

Additional file 9. Primer pairs for validation of PA clusters in 3' UTR

Gene name in RefSeq	Upstream primer	Junction primer	noT primer
KPNA4	ccaggcctacctatcatcct	ttttttttttt attatcacaagcatt	attatcacaagcatt
ITGB5	gggcaaatacgaacagattg	ttttttttttt ttccaaaattacattt	ttccaaaattacattt
CDC42EP3	gcactatttcatccagcaagc	ttttttttttt tagttgtaaaacagattt	tagttgtaaaacagattt
PCCB	acaccatgggtgcttcttt	ttttttttttt gaactttagaaggttt	gaactttagaaggttt
ACOX3	gaccgaaatgccacctgtat	ttttttttttt gaaaagatgagaaacga	gaaaagatgagaaacga
FOSL2	gcactaaagaaagcttgtta	ttttttttttt caatcttaattcatttt	caatcttaattcatttt
NDUFB5	cagataaactcaagtcgcaaaa	ttttttttttt ggggttcaggagaata	ggggttcaggagaata
CMBL	tcagagcctcacaagaatgc	ttttttttttt tcaatccacagaggt	tcaatccacagaggt
STAG1	atgccacacaaaaaggctaa	ttttttttttt attttgaatagttta	attttgaatagttta
RAD54L2	gtgtgatcatgggcttcc	ttttttttttt gagtttaaattttatttta	gagtttaaattttatttta
