# Supplementary material for: Distinct polyadenylation landscapes of diverse human tissues revealed by a modified PA-seq strategy
Source: BMC Genomics. 2013 Sep 11;14:615. doi: 10.1186/1471-2164-14-615 (PMC3848854; doi:10.1186/1471-2164-14-615)
Supplement: Additional file 10 — Primer pairs for validation of PA clusters in intronic regions. [file 1471-2164-14-615-S10.pdf]

Additional file 10. Primer pairs for validation of PA clusters in intronic regions

Gene name in RefSeq	Upstream primer	Junction primer	noT primer
MTHFD1L	caatcgtacttgtgccgaga	ttttttttttt ggctgtttttacattt	ggctgtttttacattt
ZNF518B	ggcaagtgagctcaaaatca	ttttttttttt tggtcaataaataag	tggtcaataaataag
ASAH1(5')	gcacaggtgcacaggtctta	ttttttttttt atattgttcagttctatt	atattgttcagttctatt
KIAA0141	ggttagatgggtttgggaaa	ttttttttttt tacagttcacgcatatat	tacagttcacgcatatat
COL14A1	gatgggcatctttctccttg	ttttttttttt aaatattatgttttatttac	aaatattatgttttatttac
RPLP1	ggtccttccgaggaagctaa	ttttttttttt tgaccctttttggga	tgaccctttttggga
ASAH1(3')	tgcccctttgcagaaaatac	ttttttttttt actacaatatttata	actacaatatttata
CCDC109B	ttggtatgggctattccatgt	ttttttttttt ccatctaaacccatac	ccatctaaacccatac
LHFPL3	cctgagccaaagaaacctga	ttttttttttt caggagatggtgcgt	caggagatggtgcgt
PCMT1	gtgaaagggtgtggatttta	ttttttttttt caacatttttcacattt	caacatttttcacattt
SLC25A37	ccctgacctacccctgattc	ttttttttttt gactcttcagcaatg	gactcttcagcaatg
