# Supplementary material for: Distinct polyadenylation landscapes of diverse human tissues revealed by a modified PA-seq strategy
Source: BMC Genomics. 2013 Sep 11;14:615. doi: 10.1186/1471-2164-14-615 (PMC3848854; doi:10.1186/1471-2164-14-615)

Additional file 11. Enrichment of UGUA and UGUG motifs in NP/BP and WP PA clusters.

Two 4-mer motifs, UGUA and UGUG (TGTA and TGTG at the DNA level were scanned in 100-bp regions surrounding NP/BP and WP clusters. We evenly divided the 100 bp regions into 20 bins (x axis) and the motif frequencies (y axis) of individual bins were plotted. The "0" position denotes the mode of the PA cluster.

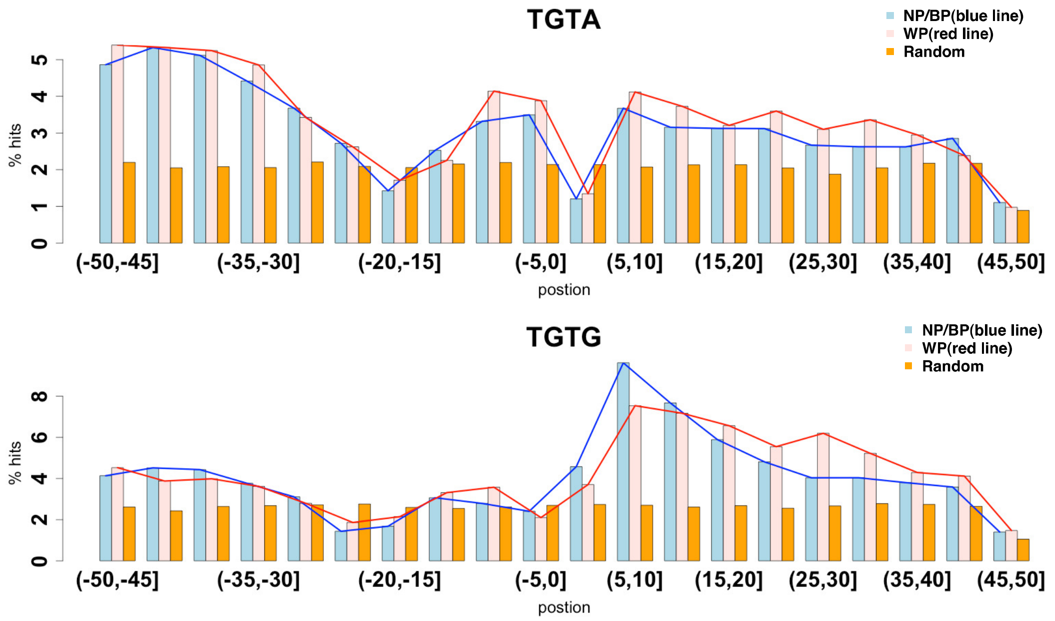

Supplement: Additional file 11 — Enrichment of UGUA and UGUG motifs in NP/BP and WP PA clusters. Two 4-mer motifs, UGUA and UGUG (TGTA and TGTG at the DNA level were scanned in 100-bp regions surrounding NP/BP and WP clusters. We evenly divided the 100 bp regions into 20 bins (X-axis) and the motif frequencies (Y-axis) of individual bins were plotted. The “0” position denotes the mode of the PA cluster. [file 1471-2164-14-615-S11.pdf]
