# Supplementary material for: Distinct polyadenylation landscapes of diverse human tissues revealed by a modified PA-seq strategy
Source: BMC Genomics. 2013 Sep 11;14:615. doi: 10.1186/1471-2164-14-615 (PMC3848854; doi:10.1186/1471-2164-14-615)

**Additional file 12. Distinct patterns of 3' UTR shortening/lengthening in human tissues.** Upper panel, genes with lower expression rank and longer 3' UTR are enriched in specified tissue (Kidney and Liver); Middle panel, genes with higher expression rank and shorter 3' UTR are enriched in specified tissue (Testis, Lung and Breast); Bottom panel, both lowly expressed genes with longer 3' UTR and highly expressed genes with shorter 3' UTR are enriched in specified tissue (Pancreas and Colon). X axis reflects the expression ranking among 13 tissues for each gene. Y axis denotes the ranking of average 3' UTR length. Color bar shows the enrichment of genes in each tissue. Red denotes enrichment while blue represents depletion.

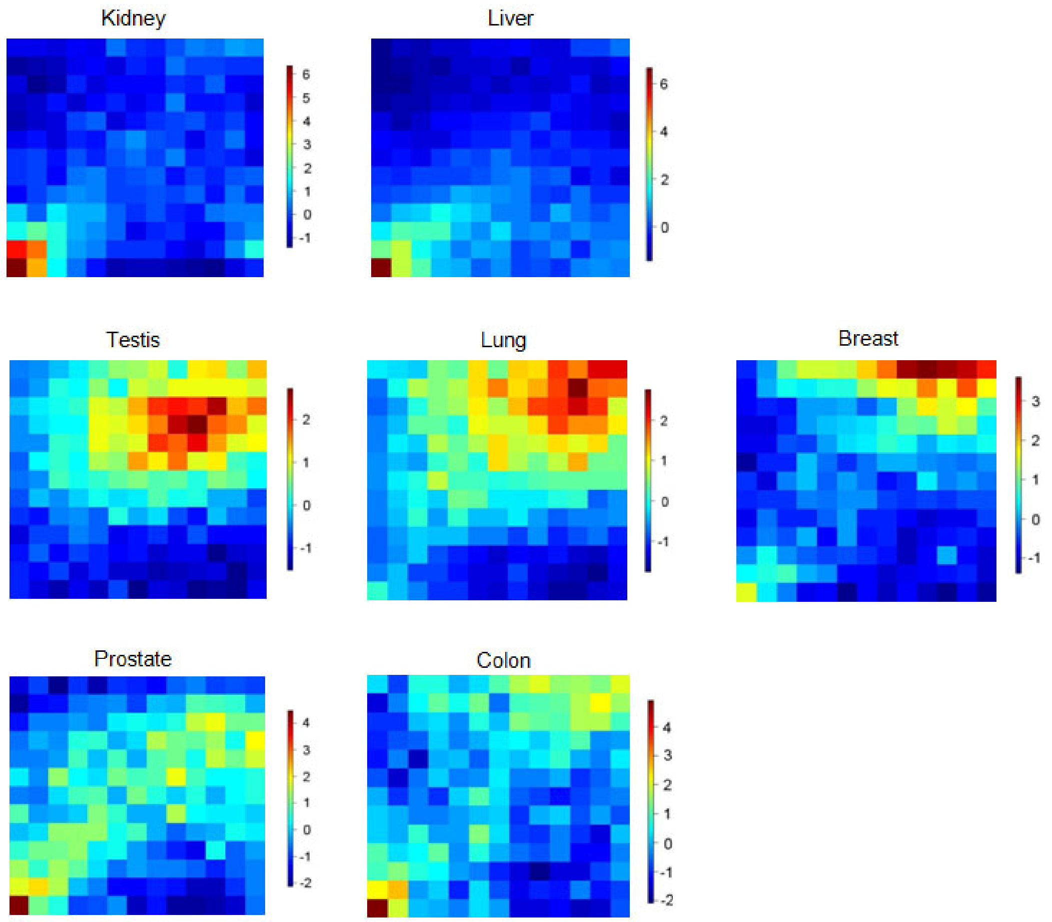

Supplement: Additional file 12 — Distinct patterns of 3′ UTR shortening/lengthening in human tissues. Upper panel, genes with lower expression rank and longer 3′ UTR are enriched in specified tissue (Kidney and Liver); Middle panel, genes with higher expression rank and shorter 3′ UTR are enriched in specified tissue (Testis, Lung and Breast); Bottom panel, both lowly expressed genes with longer 3′ UTR and highly expressed genes with shorter 3′ UTR are enriched in specified tissue (Pancreas and Colon). X-axis reflects the expression ranking among 13 tissues for each gene. Y-axis denotes the ranking of average 3′ UTR length. Color bar shows the enrichment of genes in each tissue. Red denotes enrichment while blue represents depletion. [file 1471-2164-14-615-S12.pdf]
