# Supplementary material for: Distinct polyadenylation landscapes of diverse human tissues revealed by a modified PA-seq strategy
Source: BMC Genomics. 2013 Sep 11;14:615. doi: 10.1186/1471-2164-14-615 (PMC3848854; doi:10.1186/1471-2164-14-615)
Supplement: Additional file 13 — GO analysis of genes (top right corner in dashed square) in Adult Brain. [file 1471-2164-14-615-S13.pdf]

Additional file 13. GO analysis of genes (top right corner in dashed square) in Adult Brain

Category	Term	Count	%	P Value
GOTERM_CC_FAT	GO:0045202~synapse	49	6.843575	2.15E-14
GOTERM_CC_FAT	GO:0044456~synapse part	37	5.167598	4.33E-12
GOTERM_BP_FAT	GO:0007268~synaptic transmission	38	5.307263	6.33E-11
GOTERM_BP_FAT	GO:0019226~transmission of nerve impulse	41	5.726257	1.36E-10
GOTERM_CC_FAT	GO:0043005~neuron projection	39	5.446927	3.99E-09
GOTERM_BP_FAT	GO:0050877~neurological system process	83	11.59218	2.32E-08
GOTERM_BP_FAT	GO:0007616~long-term memory	8	1.117318	5.61E-08
GOTERM_CC_FAT	GO:0005886~plasma membrane	201	28.07263	6.70E-08
GOTERM_MF_FAT	GO:0022836~gated channel activity	33	4.608939	2.17E-07
GOTERM_MF_FAT	GO:0015267~channel activity	39	5.446927	2.97E-07
