# Supplementary material for: Distinct polyadenylation landscapes of diverse human tissues revealed by a modified PA-seq strategy
Source: BMC Genomics. 2013 Sep 11;14:615. doi: 10.1186/1471-2164-14-615 (PMC3848854; doi:10.1186/1471-2164-14-615)
Supplement: Additional file 14 — GO analysis of genes (top right corner in dashed square) in Fetal Brain. [file 1471-2164-14-615-S14.pdf]

Additional file 14. GO analysis of genes (top right corner in dashed square) in Fetal Brain

Category	Term	Count	%	P Value
GOTERM_BP_FAT	GO:0019226~transmission of nerve impulse	39	5.454545	1.39E-09
GOTERM_BP_FAT	GO:0007268~synaptic transmission	35	4.895105	2.95E-09
GOTERM_CC_FAT	GO:0045202~synapse	39	5.454545	5.14E-09
GOTERM_CC_FAT	GO:0044456~synapse part	31	4.335664	1.16E-08
GOTERM_MF_FAT	GO:0022836~gated channel activity	34	4.755245	1.81E-08
GOTERM_BP_FAT	GO:0030182~neuron differentiation	42	5.874126	2.40E-08
GOTERM_BP_FAT	GO:0007409~axonogenesis	26	3.636364	3.14E-08
GOTERM_CC_FAT	GO:0034702~ion channel complex	27	3.776224	5.01E-08
GOTERM_MF_FAT	GO:0022838~substrate specific channel activity	38	5.314685	8.84E-08
GOTERM_MF_FAT	GO:0005216~ion channel activity	37	5.174825	1.23E-07
