# Supplementary material for: Distinct polyadenylation landscapes of diverse human tissues revealed by a modified PA-seq strategy
Source: BMC Genomics. 2013 Sep 11;14:615. doi: 10.1186/1471-2164-14-615 (PMC3848854; doi:10.1186/1471-2164-14-615)
Supplement: Additional file 15 — Mapping quality comparison between our data and data published by Derti et al. [file 1471-2164-14-615-S15.pdf]

Additional file 15. Mapping quality comparison between our data and data published by Derti et al.

Sample	Raw reads	Mapped reads	% of Mapped reads	Uniquely mapped reads	% of Uniquely mapped reads
Dr. Derti's kidney Study (SRR299107)[1]	18,236,241	11,740,337	64.38%	7,985,648	43.79%
Dr. Derti's kidney Study (SRR299107)[2]	18,236,241	12,749,225	69.91%	8,574,479	47.19%
Dr. Derti's liver Study (SRR299108)[3]	17,871,073	10,934,079	61.18%	7,829,366	43.81%
Dr. Derti's brain Study (SRR299106)[4]	18,540,664	9,012,532	48.61%	6,972,154	37.60%
Our data Kidney (read 1)[5]	8,049,781	7,224,707	89.75%	6,258,757	77.75%
Our data Kidney (Paired reads)[6]	8,049,781	7,244,408	89.99%	6,857,322	85.19%
Our data Liver (read 1)[7]	2,853,453	2,558,627	89.67%	2,140,902	75.03%
Our data Adult_brain (read 1)[8]	8,210,666	7,180,793	87.46%	4,938,379	60.15%

[1,3,4] bwa aligner with maximum 2 mismatches for single end reads.

[2] bwa aligner with maximum 4 mismatches for single end reads.

[5,7,8] bwa aligner with maximum 2 mismatches for single end reads. Raw reads mean after barcode split and removal of reads with TTT.

[6] bwa aligner with maximum 2 mismatches for paired end reads. Raw reads mean after barcode split and removal of reads with TTT in both read 1 and read 2.
