# Supplementary material for: Distinct polyadenylation landscapes of diverse human tissues revealed by a modified PA-seq strategy
Source: BMC Genomics. 2013 Sep 11;14:615. doi: 10.1186/1471-2164-14-615 (PMC3848854; doi:10.1186/1471-2164-14-615)
Supplement: Additional file 16 — Comparison of PA peaks identified by PolyA-seq (Derti) and PA-seq (this study). [file 1471-2164-14-615-S16.pdf]

Additional file 16. Comparison of PA peaks identified by PolyA-seq (Derti) and PA-seq (this study).

	**Peaks ≥ 50 tags***	**Peaks that overlap RefSeq annotation****
**Our Kidney Dataset**	6,646	3,914 (58.9%)
**Derti's Kidney Dataset (SRR299107)**	7,606	3,510 (46.1%)

* The starting bed file for F-seq peak calling is based on bwa mapping allowing maximum 2 mismatches (see Additional Table 6 for details). We used paired-end mapping for out kidney dataset and single end mapping for Derti's kidney dataset.

** The percentage of peaks overlap RefSeq annotation (within ±5bp of known PA sites) was indicated in parentheses.
