# Supplementary material for: Distinct polyadenylation landscapes of diverse human tissues revealed by a modified PA-seq strategy
Source: BMC Genomics. 2013 Sep 11;14:615. doi: 10.1186/1471-2164-14-615 (PMC3848854; doi:10.1186/1471-2164-14-615)
Supplement: Additional file 18 — A step-by-step protocol of PA-seq. [file 1471-2164-14-615-S18.pdf]

Additional file 18. A step-by-step protocol of PA-seq

Fragmentation and Reverse transcription

10 μg of total RNA (DNA-free) was dissolved in 30 μl fragmentation buffer (40 mM Tris-HAc (pH 8.2), 100 mM KAc and 30 mM $MgAc_2$) and heated at 94°C for 3 min. RNA fragments were precipitated with GlycoBlue (Ambion) as a carrier. Reverse transcription (RT) of the recovered RNA was performed with SuperScript II reverse transcriptase (Invitrogen) in a 50 μl reaction, containing 10 pmol oligo(dT) primer (5'-bio–TTTTTTTTTTTTTTTT dUTTTVN-3'), 100 units of RNasin (Promega) and 6 ng/μl freshly-made actinomycin D (which inhibits DNA-dependent DNA polymerase activity of reverse transcriptase). RT reaction was incubated at 42°C for 2 min before adding reverse transcriptase. We then incubate the reaction at 42°C for 60 min and 75°C for 15 min. First-stand cDNAs were then purified by ZYMO clean & concentrator-5 kit.

Second strand synthesis

Second-strand synthesis was carried out in a 50 μl reaction, containing 1x 2nd-stand buffer (500 mM Tris-HCl, pH7.8, 50 mM MgCl2 and 10 mM DTT), 40.5 μl cDNA and 15 pmol dNTP. After incubation on ice for 5 min, 25 units of DNA polymerase I (NEB) and 1 unit of RNase H (Invitrogen) were added, followed by incubation at 15°C for 2.5 hours.

Pull down of dsDNA with magnetic beads

50 μl Dynabeads MyOne C1 (Invitrogen) magnetic beads were used to pull down each sample according to vendor's protocol. Resuspend beads in 44 μl 10 mM Tris-HCl (pH7.4). 1 μl APex Heat-Labile Alkaline Phosphatase (Epicentre) and 5 μl 10x Apex buffer (Epicentre) were added to the 44 μl beads. We incubate the reaction at 37 °C for 10 min, followed by heating at 70 °C to inactivate the heat-labile Alkaline Phosphatase. The beads were then washed twice with 300 μl 1x binding & washing buffer and once with 300 μl 10 mM Tris-HCl (pH 7.4). We resuspend the beads in 48 μl TE1 buffer (10mM Tris-HCl, 0.1mM EDTA, pH8.0).

USER enzyme digestion

2 μl of USER enzyme (NEB) was added to the 48 μl beads. Release of dsDNA was carried out by incubating at 37 °C for 1 hour. ZYMO clean & concentrator-5 kit was used to purify the dsDNA.

End repair and A-tailing

The released DNA was end repaired by 3 units T4 DNA polymerase (NEB) in 1x NEB buffer 2 and 300 μM dNTP (Bioline). The reaction was incubated at 15 °C for 15 min, followed by purification with ZYMO clean & concentrator-5 kit. Eluted DNA was A-tailed by Klenow (exo-) DNA polymerase (Epicentre) with 200 μM dATP. We incubate the reaction at 37 °C for 30 min and purify the DNA with ZYMO clean & concentrator-5 kit following manufacturer's protocol.

Y-linker ligation

Ligation was performed in a 10 µl reaction by adding 3 pmol Illumina paired-end Y-linker, 1 µl 10x T4 DNA ligase buffer (NEB) and 1 µl T4 DNA ligase (NEB; 2000 units/µl). After incubation at room temperature (or 25 °C) for 30 min, the ligation products were purified by ZYMO clean & concentrator-5 kit, followed by size-selection in a 2% agarose gel to obtain 300-400 bp DNA fragments. The gel slice was purified by ZYMO gel purification kit and elute with 20 µl nuclease-free water.

Low-cycle PCR

PA-seq library was amplified by low-cycle PCR before Illumina paired-end sequencing. Since we remove the phosphate group in upper strand by Alkaline Phosphatase, only bottom strand can be ligated and further amplified by PCR. A 50 µl reactions was assembled for each library, which contains size-selected DNA, 1x HF buffer (Finnzymes), 1 nmol dNTP, 25 pmol of the Forward primer (5'- AAT GAT ACG GCG ACC ACC GAG ATC TAC ACT CTT TCC CTA CAC GAC GCT CTT CCG ATC T-3') and the Reverse primer (5'- CAA GCA GAA GAC GGC ATA CGA GAT CGG TCT CGG CAT TCC TGC TGA ACC GCT CTT CCG ATC T-3') and 0.5 µl of Phusion Hot Start High-Fidelity DNA Polymerase (Finnzymes). Thermal cycling was carried out as the following: 98 °C for 30s; 16 cycles of 98 °C for 10s, 67 °C for 30s and 72 °C for 30s; 72 °C for 10 min; hold at 10°C. The PCR products (or final PA-seq library) were purified by ZYMO clean & concentrator-5 kit and quantified by Qubit Fluorometer (Invitrogen) before Illumina paired-end sequencing.
